# The Use of Medicinal Plant-Derived Metallic Nanoparticles in Theranostics

**DOI:** 10.3390/pharmaceutics14112437

**Published:** 2022-11-10

**Authors:** Jabulile Happiness Xulu, Tanaka Ndongwe, Kenneth M. Ezealisiji, Vuyelwa J. Tembu, Nontobeko P. Mncwangi, Bwalya A. Witika, Xavier Siwe-Noundou

**Affiliations:** 1 Department of Pharmaceutical Sciences, School of Pharmacy, Sefako Makgatho Health Sciences University, Pretoria 0204, South Africa; 2 Department of Pharmaceutical and Medicinal Chemistry, Faculty of Pharmaceutical Sciences, University of Port Harcourt, PMB 5323 Choba, Rivers State, Nigeria; 3 Department of Chemistry, Tshwane University of Technology, Pretoria 0001, South Africa; 4 Department of Pharmacy Practice, School of Pharmacy, Sefako Makgatho Health Sciences University, MEDUNSA, Pretoria 0204, South Africa

**Keywords:** theranostics, metallic nanoparticles, medicinal plants, phytochemicals, cancer, malaria, microbial, cardiovascular diseases

## Abstract

In the quest to effectively diagnose and treat the diseases that afflict mankind, the development of a tool capable of simultaneous detection and treatment would provide a significant cornerstone for the survival and control of these diseases. Theranostics denotes a portmanteau of therapeutics and diagnostics which simultaneously detect and treat ailments. Research advances have initiated the advent of theranostics in modern medicine. Overall, theranostics are drug delivery systems with molecular or targeted imaging agents integrated into their structure. The application of theranostics is rising exponentially due to the urgent need for treatments that can be utilized for diagnostic imaging as an aid in precision and personalised medicine. Subsequently, the emergence of nanobiotechnology and the green synthesis of metallic nanoparticles (MNPs) has provided one such avenue for nanoscale development and research. Of interest is the drastic rise in the use of medicinal plants in the synthesis of MNPs which have been reported to be potentially effective in the diagnosis and treatment of diseases. At present, medicinal plant-derived MNPs have been cited to have broad pharmacological applications and have been studied for their potential use in the treatment and management of cancer, malaria, microbial and cardiovascular diseases. The subject of this article regards the role of medicinal plants in the synthesis of MNPs and the potential role of MNPs in the field of theranostics.

## 1. Introduction

The use of theranostics has ignited particular interest in modern science. In recent years, researchers have sought to exploit the potential use of theranostics in numerous fields. Theranostics is a broad field of science that aims to develop diverse and intricate diagnostic and therapeutic agents [1,2]. It is also established that these cutting-edge systems, when coupled into a single platform, are capable of bridging the gap between imaging molecules and therapeutic agents with regard to biodistribution and site specificity [1,2,3]. Presently, theranostics, through the use of nanotechnology techniques, deliver active pharmaceutical ingredients (APIs) to the absorption sites, resulting in increased bioavailability. In addition to the aforementioned advantages, theranostics have been proposed to be potentially effective in various ailments, particularly in cancer, malaria, microbial diseases as well as cardiovascular diseases by using MNPs [4,5]. Moreover, theranostics are essential in personalised medicine as they can be developed on the basis of biomarker identification [6]. The versatility of MPNs allows them to be some of the most promising diagnostic and therapeutic entities in modern medicine.

Recently, there has been a drastic rise in the biosynthesis of MNPs from medicinal plants that are essential in the development of theranostics. Medicinal plants serve as a reliable and an indispensable source of natural bioactive compounds. It has been reported that ~80% of the world’s population is still dependent on the use of medicinal plants for their primary healthcare and for the development of a myriad of medicines [7]. Currently, over 40% of pharmaceutical formulations are derived from natural ingredients and include commercially available medicines such as digoxin, chloroquine quinine, lumefantrine, atovaquone, aspirin and artemisinin [8,9].

Nanoparticles (NPs) are materials with the longest dimension of <100 nm, with MNPs being specifically composed of metals as the primary material [10,11]. The use of NPs has brought notable improvements in nanomedicine, especially in reducing the dosing frequency, improving the solubility of drugs and increasing the half-life of some drugs; this has resulted in commendable changes in targeted drug delivery [3,12,13]. NPs have also been reported to be more selective and sensitive in the diagnosis of diseases, especially in cancer. Recent advances in nanomedicine include the incorporation of nano-vehicles for optimal drug delivery using biosynthesized MNPs [4,5,13,14]. By doing so, research on the biosynthesis of MNP using medicinal plants is drawing considerable attention as an emerging branch of science.

Medicinal plants serve as a source of phytochemicals that can be used to substitute chemical-reducing agents such as sodium citrate, sodium borohydride and ascorbate, which are very toxic, expensive, and in many cases, damaging to the environment [4,11,13,15]. Consequently, multiple physicochemical approaches have been utilised to engineer MNPs including the use of medicinal plant parts such as leaves, fruits, stems, roots and seeds in a cost-effective way [11]. It has been demonstrated that phytochemicals from plant extracts, such as polysaccharides, flavonoids, phenolic acids and alkaloids, are capable of effectively reducing metal ions, such as Ag^+^, Cu^2+^ and Au^3+^ [11,13,16]. Moreover, during the formation of NPs, phytochemicals play a pivotal role in the capping, stabilising and chelating of NPs. This makes phytochemicals ideal entities in the biosynthesis of MNPs [16]. It is also underscored that the biosynthesis of MNPs with medicinal plants results in the improvement of the safety profile of theranostics agents because of the decrease in the anticipated toxicity [11,13,16]. Traditional physical and chemical procedures used in the manufacturing of MNPs have been reported to be more labour-intensive and toxic. On the other hand, biologically-mediated synthesis, using a variety of biological systems including bacteria, fungi and medicinal plant extracts, might produce large quantities of MNPs with specific sizes faster, that are safer and more sustainable [17,18,19]. In this review, focus is placed not only on the present contemporary medicinal plant-derived MNPs, but also on highlighting the gaps in the field of theranostics.

## 2. Medicinal Plant-Derived Phytochemicals Used in the Green Synthesis of MNPs

Green nanotechnology is derived from green chemistry which aims to produce suitable phytoformulations [20]. The versatility of green nanotechnology has extended to the synthesis of NPs and nanoproducts which have immensely contributed to the environmental sustainability [16,21]. The interest lies in the use of medicinal plants in nanoparticle formulation because they are easily accessible and offer a wide variety of metabolites that are essential in the formulation of NPs [22]. In this regard, green nanotechnology utilises medicinal plants to synthesise nanomaterials including MNPs that may be potentially effective in the diagnosis and treatment of many diseases. The use of medicinal plant-derived MNPs is considered less hazardous and is relatively cheap [13,23,24]. Furthermore, the literature suggests that the sizes and shapes of plant-derived MNPs can be modified to meet the desired formulations. Notwithstanding the benefits associated with the use of plant-derived MNPs, their safety profile is still questionable. MNPs have been reported to have a low biocompatibility, which makes them difficult to formulate in the desired medium [24]. Moreover, some MNPs have a low biodegradability and may lead to cumulative toxicity [25,26].

The role of medicinal plants in modern medicine is essential as they serve as a reliable source of diverse and numerous chemical entities that are essential in the biosynthesis of MNPs [24,27,28]. The biosynthesis of MNPs involves the use of medicinal plant-derived phytochemicals such as alkaloids, flavonoids, saponins, tannins, phenols and terpenoids. These compounds are used as reducing, capping and stabilizing agents that interact with NPs through the reduction of MNPs [22,29]. In this regard, researchers are seeking to explore phytochemicals that will also collectively work together with MNPs in the management of diseases. Currently, a considerable number of compounds especially phytochemicals have been investigated for their potential role in the biosynthesis of MNPs. These compounds are either reported as part of plant extracts or as pure compounds and some of the phytochemicals are depicted in Table 1.

Despite the notable advances in the biosynthesis of MNPs, marine plants have not been fully explored in the synthesis of MNPs. Currently, metallic moieties such as Ag, Au, cadmium (Cd) and titanium oxide (TiO_2_) have been reported in the biosynthesis of MNPs through the use of marine plants. Examples of some of the leading marine plants that have been investigated in the biosynthesis of MNPs are viz. *Rhizophora mucronate*, *Avicennia marina*, *Prosopis chilensi*, *Citrullus colosynthis*, *Sargassum ilicifolium*, *Xylocarpus mekongensis*, *Cymodocea serrulata*, *Syringodium isoetifolium*, *Sargassum myriocystum* and *Enhalus acoroides* [66,67].

**Figure 1:**
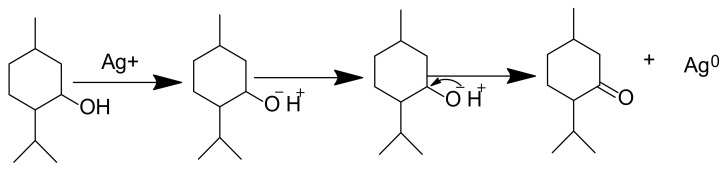
Adapted with permission from [76]. Copyright (2022) Elsevier Heliyon. Phyto-reduction of Ag^+^ to AgNPs by terpenoids.

Parts of medicinal plants that are predominantly used in the green synthesis of MNPs are leaves, flowers, roots, stem bark and fruits which are added to an aqueous solution of metal ions to begin the biosynthesis process. Flavonoid, phenols, terpenoids and organic acids are among the phytochemicals found in medicinal plant extracts that are mostly used as stabilizing and reducing agents [68,69,70]. Capping agents are known to stabilize NPs by hindering the agglomeration of the NPs [71]. Moreover, capping agents also play a vital role in influencing the morphology of nanostructures [72]. It is also suggested that the molecular weight (MW) of phytochemicals used as capping agents greatly influences the nanoparticle assembly behaviour, as this is also affects the van der Waals interaction, capillary interaction and the effect of the hydrogen bond [68]. A reducing agent converts metal ions to nanometal during the synthesis of nanoparticles, particularly MNPs [73]. However, some reducing chemicals are known to interact with stabilizer molecules—this then solves the problem of reducing and capping at the same time [74,75]. The reduction mechanism of Ag ion (Ag^+^), known as state one to (Ag^0^) state zero by the phytochemical terpenoid, is depicted in Figure 1.

The biosynthesis approach necessitates a thorough comprehension of the raw materials such as plant extracts, particularly in relation to their synthesis into nanometals [11]. Ultimately, the employment of biosynthesis methods, such as reducing, capping and stabilizing agents in the synthesis of MNPs, has risen drastically [16]. To choose the best organisms for extract synthesis, one must consider the metabolic pathways, phytochemical content, enzyme activity, cell proliferation and appropriate reaction conditions [76]. There is an urgent need for clean, dependable and eco-friendly approaches to counteract the already known hazardous methods as they mostly use toxic material [77,78]. As a result, green synthesis methodologies based on medicinal plant extracts, microorganisms and some marine algae have emerged as eco-friendly nanoparticle manufacturing methods [78]. In recent times, biological techniques have offered a superior platform for the synthesis of MNPs such as AgNPs. In comparison to the chemical and physical approaches, the green synthesis method has the most advantages since it is economical, environmentally friendly and easy to scale up for large-scale synthesis without using energy, high pressure, high temperature or harmful chemicals [79,80,81].

**Figure 2:**
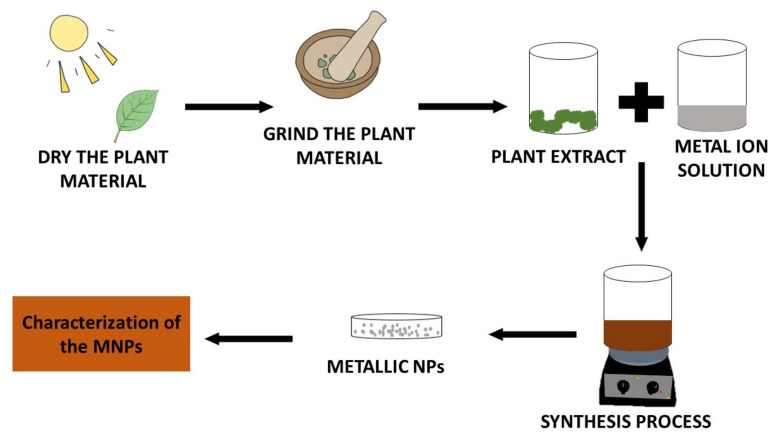
The stepwise flow for the synthesis of MNPs using medicinal plant extract.

A stepwise process commonly followed when the green synthesis of MNPs is utilised is depicted in Figure 2.

## 3. Synthesis of Metallic Nanoparticles (MNPs)

**Figure 3:**
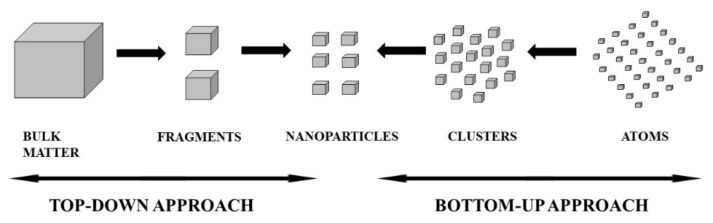
A summary of the general techniques utilised in the manufacturing of MNPs.

The choice of the preparation method for MNPs is important during nanoparticle synthesis [76,77]. Physical and chemical synthesis techniques are known to be potentially toxic, and frequently expensive compounds are utilized in the synthesis and stabilization of the MNPs which result in by-products that are not good for the environment [81,82]. Factors such as kinetics of interaction of the metal ions with a reducing agent, absorption process of stabilizing agent with MNPs and varied experimental techniques produce a significant impact on its morphology stability and physicochemical properties of the NPs [17,78]. The synthesis of MNPs involves many methods which can be divided into two general categories viz., bottom-up methods and top-down approaches [69]. The top-down approach is the process in which bulk matter is broken by physical methods i.e., pulverization until it is a small nanoparticle size. In the bottom-up approach, small atom-sized matter is built up using chemical methods i.e., chemical reduction until NPs are synthesized. In the process of synthesising MNPs through physical and chemical techniques, the breakdown technique (top-down) is commonly preferred. In the top-down approach, which is frequently referred to as the mechanochemical method, physical forces including grinding, pulverization and other methods are utilised to reduce the size of bulk material as a precursor to the nanosize [83,84]. The bottom-up strategy incorporates the coalescence or assembly of atoms by atoms, molecules by molecules and clusters by clusters, resulting in a yield with a variety of NPs. The synthesis of NPs, and diverse and numerous techniques are used, including plasma or flame spraying, chemical vapor deposition (CVD), sol-gel processing, the self-assembly of both monomer and polymer molecules, chemical nanostructural precipitation, laser pyrolysis and bio-assisted synthesis [83,85]. Additionally, the use of toxic chemicals and reagents in the synthesis process produces toxins that are not environmentally friendly, which is why green chemistry is gaining popularity because it is known to be less toxic [18,67,86]. The bottom-up and top-down approaches are summarised in Figure 3.

## 4. Characterisation of MNPs

To gain insight into the physico-chemical behaviours of nanomaterials that will influence their pharmacological profile and the precision of the NPs, it is crucial to comprehend how distinct physicochemical features of NPs influence their in vivo distribution and behaviour. This necessitates the use of dependable and durable methods for evaluating the various physicochemical properties of nanomaterials in general and nanomedicine in particular [87]. A rigorous yet practical approach to the trustworthy characterisation of nanomaterials is crucial for the safe, rational development of nanomedicines and theranostics [88,89]. The analytical techniques that are used to determine the physiochemical properties of NPs are described in Table 2.

## 5. Therapeutic and Diagnostic Applications of MNPs

### 5.1. MNPs in Cancer

Cancer is one of the leading fatal diseases that claims the lives of 70% of people globally and is mostly prevalent in middle- and low-income countries. The onset of cancer is triggered by both external and internal factors [109,110,111,112,113]. Researchers have made an effort to look into how plant-derived NPs may be related to cancer. To date, there are many traditional anticancer medications accessible, but the majority of them are associated with genotoxicity, teratogenicity and carcinogenicity [112,113,114]. Contrary to their considerable effectiveness against malignant cells, the adverse effects of anticancer medications have restricted their use in the treatment of cancer. As a result, scientists are paying closer attention to discovering novel bioactive plant compounds that are both efficient and safe [109,110,111,112,113,114].

A study by Jain et al. aimed to design AgNPs using three species of *Curcuma*—namely, *Curcuma aromatic*, *Curcuma longa* and *Curcuma caesia*—for the treatment of human colon cancer. The AgNPs were synthesized using the rhizomes, and the plant species were mixed with silver nitrate (AgNO_3_) and maintained at 37 °C. The sulforhodamine B (SRB) assay demonstrated that, to varying degrees, the AgNPs of *C. caesia*, *C. longa* and *C. aromatica* reduced the percentage viability of HT-29 human colon cancer cells. It was then concluded from the study that the synthesized AgNPs of *Curcuma* extract significantly inhibited the growth of HT-29 colon cancer cells, thus, showing its anticancer effect [109].

Hailan et al. conducted a study where they investigated the reactive oxygen species-mediated cytotoxicity in liver carcinoma cells induced by biosynthesized AgNPs from the *Schinus molle* extract. Within 20 min of adding the *S. molle* leaf extract, the reaction mixture’s colour changed from colourless to brown, signalling the start of AgNPs synthesis. This demonstrated the AgNPs’ biogenesis. Various methods, including UV–vis spectroscopy, were used to emphasize the reduction of the Ag ions to AgNPs. The production of oxidative stress, cytotoxicity, apoptosis and autophagy by the NPs was found to suppress the proliferation of HepG2 liver cancer cells. This innovative strategy might motivate scientists to combine the efficacy of AgNPs and the potential of natural products for the development of liver cancer treatments [115].

### 5.2. MNPs in Microbial Diseases

For centuries, elemental metals such as Ag and Au have been extensively employed as antibacterials [116,117,118]. Through the use of biological methods of synthesis, MNPs such as AgNPs and AuNPs can be synthesized from plant extracts to increase their antibacterial activity and decrease their toxicity [17].

Antimicrobial resistance (AMR) is a recurrent health concern in which bacteria, viruses, fungi and parasites evolve over time and cease to respond to antimicrobial therapy, making infections more difficult to cure and raising the risk of disease transmission, life-threatening sickness and death [119]. Methicillin-resistant *Staphylococcus aureus* (MRSA) infections increase the risk of death by 64% compared to drug-sensitive infections [119]. The aforementioned statement is concerning, considering that the human epidermis is home to the bacterium *Staphylococcus aureus*, which is also a frequent source of infections in both the general population and healthcare settings [120].

Lead oxide (PbO) NPs are some of the leading compounds used in nanomedicine due to their nontoxic, biocompatible and stable effects. Shahid et al. went on to investigate the effect of green synthesis of PbONPs under ambient conditions with *Eucalyptus globulus* leaf extract as a potential nanotheranostic agent [116]. The leaf extract from *E. globulus* was prepared using a Soxhlet extractor, and lead acetate was used as a reducing and capping agent. Additionally, the precipitates were centrifuged, filtered, dried and characterised. The resultant formulation was evaluated for its antibacterial activity against *Staphylococcus aureus* and *Escherichia coli*. The PbO-NPs from *E. globulus* also showed significant antibacterial action against both Gram-positive and Gram-negative bacteria, with the largest zones of inhibition measured at 19 mm (*S. aureus*) from n-hexane extract and 16 mm (*E. coli*) from methanol extract. The study highlighted that the green synthesis of PbONPs using *E. globulus* can be considered for antibacterial use [116].

Plant-derived AuNPs are known to significantly inhibit the growth of medically important pathogenic bacteria and fungi, which makes this metal sought after for the use of antimicrobial-resistant strains [4]. AgNPs produced through green synthesis can be used to carry oligonucleotide-based antimicrobials. The AgNPs can further be encapsulated in macro-matrixes such as cyclodextrin complexes, lipid-based formulations and hydrogel nanocompsoites with the potential of offering controlled release and/or targeted delivery [121]. For the biogenic production of AgNPs, a study by Attallah et al. employed *Gardenia thailandica* leaf extract (GTLE) AgNPs. The in vivo test was carried out on rats with wounds infected with *Staphylococcus aureus* bacteria. AgNPs caused epidermal regeneration and a decrease in inflammatory cell infiltration. As a result, GTLE can be considered for the biosynthesis of AgNPs, as it has shown to be potentially effective in inhibiting *S. aureus* bacteria action in vivo and in vitro [122].

### 5.3. MNPs in Cardiovascular Diseases (CVDs)

According to the World Health Organization (WHO), more than 18.6 million fatalities worldwide occurred in 2019 as a result of CVDs, accounting for more than 31% of all deaths [123]. The development of efficient non-invasive imaging technologies for early detection and to monitor subsequent therapeutic responses to CVD treatment has become a key priority to deal with such a dire condition. The traditional treatment options include drug therapy and invasive operations such as angioplasty, stenting and bypass grafting [124,125]. The major limitations of most traditional techniques predominantly used in diagnosis and treatment have been reported to have low sensitivity, site specificity, systemic side effects, quick medication clearance and non-targeted localisation [126]. MNPs have been widely used in molecular imaging and cardiac assays of CVDs due to their good pharmacokinetic and biodistribution properties [127,128]. They can deliver enormous volumes of contrast materials in concentrated bundles to magnify signals or offer adaptable platforms for the integration of numerous functional entities. Ischemic heart disease occurs as a result of myocardial infarction (MI) [129]. Numerous drugs have been used to treat MI through a variety of mechanisms, including thrombus dissolution and myocyte healing in the infarcted area [130]. Recent investigations have revealed a novel treatment for MI utilizing MNPs. Due to their biocompatibility and numerous benefits in treating particular CVDs, AuNPs can be employed in the diagnosis and treatment of MI illness [131,132].

A study by Dong et al. aimed to combine, classify and test the effectiveness of AuNPs in inducing cardiomyoblast hypertrophy. The *Imperata cylindrica* extracts (IPC) and gold solution (HAuCl4) used in the fabrication of AuNPs were differentiated from one another by a number of characterization procedures. The rat cardiomyoblast cell lines H9c2 and 3T3 fluorescent were examined. Since more AuNPs were present during the incubation period, less superoxide anion was produced intracellularly. Additionally, preliminary results provided information on the involvement of AuNPs in reducing isoproterenol-induced cardiomyoblastic hypertrophy [133]. In another study, the *Calendula officinalis* extract was used as a stabilizing and reducing agent to form iron nanoparticles (FeNPs) in an aqueous media. DNA fragmentation and apoptosis were demonstrated using the TUNEL test, and DNA fragmentation was reduced by cell cutlers treated with FeNPs. This increased the potential of the mitochondrial membrane in high concentrations of HDMVECn, HUVEC, HAEC, HCAEC, HCASMC and HPAEC cells treated with mitoxantrone. The high dose of FeNPs was found to be 4 µg —this showed the strongest cardiovascular protective characteristics [134]. Following approval in human clinical trial research, FeNPs containing a *C. officinalis* leaf aqueous extract may be used as a cardiovascular protective supplement for treating cardiovascular disorders [134].

### 5.4. MNPs in Malaria

Malaria is one of the most common tropical diseases which is carried by the parasitic protozoan of the genus *Plasmodium falciparum* [135,136]. Researchers frequently utilise metal oxide NPs for a variety of medical applications and some of the leading MNPs are zinc oxide (ZnO), AgNO_3_, Fe_2_O_3_, CuO and aluminum oxide (Al_2_O_3_). β–Hematin (biomarkers for malaria) can be degraded by metal oxide NPs made using chemical and microwave techniques [137,138]. Since ancient times, various microbiological illnesses have been treated using silver and silver-based medicines [139]. In some cases, AgNPs have been reported to have promising antimalarial effect especially when tested against *Plasmodium falciparum* [92,140,141,142].

Okaiyeto et al. used the aqueous leaf extract of *Salvia officinalis* to synthesize AgNPs. Then, its antiplasmodial activity against *Plasmodium falciparum* and cytotoxic effect on human cervix adenocarcinoma (HeLa) cells were examined. The synthesized AgNPs showed notable antiplasmodial capability with an IC_50_ value of 3.6 μg/mL and were found to be less cytotoxic to the HeLa cell strain. The results suggested that the AgNPs might be used as a model for the development of novel medications to treat malaria; hence, more research is required to identify and describe the effective molecules that suppress the malaria parasite [143].

Ojemaye et al. conducted a study with the aim of synthesizing, characterizing and evaluating the effectiveness of AgNPs obtained from the fruit and leaf extracts of *Crataegus ambigua* against malarial parasites. With the aid of parasite viability methods, the antimalarial effectiveness of the AgNPs was evaluated in contrast to the plant extract, which had a lower percentage inhibition and proved to be inactive against *Plasmodium falciparum*. On the other hand, the synthesized NPs from the plant’s fruit and leaves had strong antimalarial activities with an IC_50_ of 20 μg/mL. The antimalarial properties of the AgNPs demonstrated that plant components from *C. ambigua* make excellent precursors for naturally occurring antimalarial medications [144].

Another study by Gandhi et al. used the *Momordica charantia* leaf aqueous extract as a stabilizing and reducing agent to create titanium oxide NPs (TiO_2_) which were then screened against *P. falciparum*. The outcome of the study showed that biosynthesized TiO_2_-loaded NPs demonstrated significant antimalarial activity against *P. falciparum* strains(CQ-s and CQ-r) with IC_50_ of 53.42 μg/ml (CQ-s) and 59.71 μg/ml (CQ-r) [12]. Furthermore, no observable toxic effects were reported implying that plant derived TiO_2_ may be safe in the management of malaria. The plant species that have been used to synthesize MNPs through green technology for their therapeutic use are summarised in Table 3.

## 6. MNPs for Diagnosis

Seemingly, the diagnostic use of medicinal plant-derived MNPs in general is still in its infancy. Most of the studies have utilised MNPs obtained from chemical and physical synthesis with a dearth of information reported for the use of plant-derived MNPs as potential diagnosis tools [167,168,169]. *Olax scandens* were investigated to show the self-fluorescence features of Ag-Cu nanocomposites in microbial cells. From the results, the authors concluded that Ag-Cu nanocomposites demonstrated red fluorescence in bacterial cells, while no fluorescence in untreated cells was observed in Ag-Cu nanocomposites. The authors underscored the potential of a nanocomposite to cause ROS may be used in the elimination of cancer cells [167].

Due to their distinctive characteristics, traits such as the surface plasma resonance (SPR) properties of MNPs have generated a great deal of interest [169]. The size, shape, composition, optical properties and internal particle interactions of the particles as well as the dielectric characteristics of the surrounding fluids play a crucial role in the SPR of MNPs [14,168,169]. From UV to the near-infrared (NIR) region, metal nanoparticles’ SPR can be adjusted, and this prosperity can be used in biosensing. Additionally, the SPR necessitates the creation of a diversity of optical methods for the diagnosis of bacterial infections, such as colorimetric, fluorescent and nonlinear optical methods [169,170].

Other traits of interest are electrochemical and magnetic properties [170]. The magnetic traits of MNPs have been used in numerous diagnostic and analytical methods including the cancer biomarkers and nuclear magnetic resonance. The fluorescence properties of MNPs are also used in the detection of microorganisms such as bacteria. Additionally, MNPs that are closely spaced from one another exhibit an interparticle plasmonic interaction, which results in a redshifted extinction spectrum and a noticeable colour change. MNPs have an extinction coefficient that is many orders of magnitude higher than that of conventional organic dye molecules [170,171]. Through the release of metal ions, the formation of ROS or photothermal effects, MNPs have been investigated for the treatment of bacteria mostly in vitro. MNPs’ chemical makeup, size and shape have been proposed to affect the antibacterial potency and range of their actions. These results may provide insight into the creation of powerful and versatile metal-based nanomedicine for the treatment of bacterial illnesses [171]. In addition, MNPs having photothermal and photodynamic properties, like AuNPs, have a greater potential for future use in cancer diagnosis and should be further studied in vivo and in preclinical settings [169,172].

## 7. Biological Safety

MNPs have been implicated in numerous toxicity studies. This can be ascribed to their size, shape and surface change, which, despite being beneficial, still carry significant shortcomings [173,174]. Generally, the shape, concentration, structure and exposure time of MNPs have been cited to affect cell cycle processes such as endocytosis, DNA synthesis, autophagy and apoptosis [173,174,175]. The literature suggests that MNPs cause toxicity through different mechanisms such as the generation of reactive oxygen species (ROS), namely H_2_O_2_, as well as releasing ions that are toxic to cells and cause physical damage to cells [176,177].

It noteworthy that there is a dearth of information pertaining to toxicity specific to MNPs biosynthesized from plants. Moreover, the toxicity of the plant-based MNPs may vary depending on how the plant material interacts with the metal ions or the biological system [76,174]. Studies on the biosynthesis do postulate the idea that MNPs synthesized from plant materials can exemplify the current known MNP toxicity [11,16,52,76,178].

### 7.1. Organ Damage

When synthesizing MNPs, it is always important to consider the human physiological response, hence, researchers use cytotoxicity as a measure of toxicity attributed to their formulation.

An aspect of concern is the cumulative toxicity associated with the use of MNPs. This arises as MNPs have a tendency of accumulating in vital body parts such as the liver and the brain [175,176,177,179]. Accumulation of MNPs in such organs may lead to diseases such as liver cirrhosis and neurological disorder [175,179]. MNPs easily pass through the brain barrier and have been implicated in potentiating neurodegenerative diseases—this might be attributed to their small size. Moreover, the surface charge also promotes the clustering of MNPs, which may also lead to cumulative toxicity [176,180,181].

Suker et al. assessed the toxicity of anatase TiO2 NPs on rat liver and BALF biochemical alterations. There were 63 mat rats—this included the control group and the experimental groups—that underwent a four-week period of twice-weekly treatment with various concentrations of nano-TiO_2_ (size 21 nm). By using an enzyme-linked immunosorbent assay (ELISA), the levels of tumour necrosis factor (TNF) and macrophage inflammatory protein (MIP)-2 were determined in the bronchoalveolar lavage fluid (BALF) supernatants and lung homogenate, and liver tissue underwent a histological examination. The findings demonstrated that TiO_2_ NP causes several structural changes in the liver. This includes an increase in Glisson capsule thickness, increased collagen density in the portal triads, and significant infiltration of inflammatory cells into the liver [182]. This study concluded that MNPs have the potential to affect vital organs such as the liver, which is predisposed to toxicity [182,183]. The accumulation of MNPs is cited to result in an increase in inflammatory cytokines and aminotransferase enzymes, which may signal liver injury [175,183,184].

Some researchers have underscored that MNPs inhibit the electron transport chain which is essential for cell survival [185]. Moreover, MNPs with a particle size of <50 nm have been reported to be toxic to nearly all types of cells while MNPs of <100 nm are easily absorbed in the intestines and can circulate in the lymphatic system where they are likely to cause toxic effects [177]. Smaller MNPs such as AgNPs (10 nm size) have been found to be more hazardous than AgNPs, with a size range of 50 and 100 nm in inducing necrotic cell death in PC12 cells [186].

Mukherjee et al. investigated the toxicity of MNPs in female mice using AuNPs synthesized from *Peltophorum pterocarpum*. The authors reported that the AuNPs demonstrated no significant cytotoxicity when administered at 1 mg/kg and 10 mg/kg for a period of seven days [187]. No significant changes were observed after evaluating the haematology and serum biochemistry reports. On the contrary, under the same experimental circumstances, the mouse group that was given chemically produced pegylated AuNPs showed notable toxicity signs. In rat models, MNPs such as Au, TiO_2_ and Zn have been cited to accumulate in the brain parenchyma. Some studies have even reported that TiO_2_NPs impair memory and affect brain development—this again highlights the dangers directly linked to MNPs [188,189].

### 7.2. DNA Damage and Genotoxicity

Gene alteration is another factor associated with the use of MNPs; this happens through the generation of ROS which causes breakages in DNA strands and leads to changes in gene expression. Studies have also highlighted that MNPs such as Ag, CuO, Fe_2_O_3_ and TiO_2_-derived NPs can cause DNA damage partly due to the increase in ROS [190,191].

Santonastaso and co-workers investigated the genotoxic effects of TiO_2_-NPs on human spermatozoa in vitro [192]. New insights were presented on DNA damage in human sperms that were in vitro and subjected to two concentrations of n-TiO_2_ (1 g/L and 10 g/L) for varying periods of time. The outcome of the study showed a statistically significant loss of sperm DNA integrity after 30 min of exposure to MNPs. The results highlight the potential genotoxicity linked with MNPs that may result in fertilization instability [192]. In a study by Ma et al., the ICR mice were given daily injections of different dosages of nanoparticulate anatase TiO_2_ (5 nm) into the abdominal cavity for 14 days in order to assess the effects of the particles on the brain [193]. The coefficient of the brain, pathogenic alterations in the brain and oxidative stress-mediated reactions, as well as the build-up of nanoparticulate anatase TiO_2_ and levels of neurochemicals in the brain, were then investigated. The brain damage and oxidative stress occurred as a result of a cascade of events that seemed to have been initiated by nanoparticulate anatase TiO_2_. This included lipid peroxidation, decreased total anti-oxidation capacity and antioxidative enzyme activities, excessive nitric oxide release, reduced glutamic acid and downregulated levels of acetylcholinesterase activities. The authors concluded that TiO_2_ NPs injected into the abdominal cavity have the potential to reach the brain and induce brain damage [193].

Similarly, TiO_2_ NPs have been implicated in inducing cell changes and impairing enzyme functions in hFOB 1.19 cells. These nanoparticles were found to result in cell death in a time- and concentration-dependent manner [181,189].

Song et al. examined how mice exposed to nanoparticles CuO, Fe_3_O_4_, Fe_2_O_3_, TiO_2_ and Ag were affected by the induction of micronucleated reticulocyte production and oxidative stress [191]. Peripheral blood was drawn from the tail at 0, 24, 48 and 72 h following an intraperitoneal injection of nanoparticles for the micronucleus assay. The urinary 8-hydroxy-2′-deoxyguanosine levels were measured using the high-performance liquid chromatography with electrochemical detection (HPLC-ECD) method after mice were injected intraperitoneally with nanoparticles to track the oxidative stress. The results showed that development of reticulocyte micronuclei and urinary 8-hydroxy-2′-deoxyguanosine levels increased in all groups treated with nanoparticles. The dose-dependent increase in 8-hydroxy-2′-deoxyguanosine levels in the liver DNA of the CuO-treated group was observed. In conclusion, oxidative stress may be responsible for the toxicity of these MNPs, which caused genotoxicity towards the mice [191].

### 7.3. Fetotoxicity

Researchers have highlighted that MNPs including TiO_2_ pose significant safety concerns to the foetus as they are able to cross the placenta [184,194]. For example, TiO_2_NPs have been cited to impair foetal growth and resorption after crossing the placenta. Additionally, TiO_2_NPs have been reported to alter gene expression and cause notable breaks in foetal liver cells [194].

A study by Campagnolo and co-workers aimed at determining whether inhaled AgNPs can penetrate the mouse placental barrier and cause undesired effects. During the first 15 days of gestation, the mice were exposed via nose-only inhalation to a newly formulated aerosol of 18–20 nm AgNPs for 1–4 h, depending on the nanoparticle concentration. During the first 4 h of the day, there was a rise in the number of resorbed embryos and a decrease in oestrogen plasma levels in the exposed mice. The placentas in both groups were found to express pregnancy-relevant inflammatory cytokines more frequently. These findings demonstrate that NPs can enter and pass through the mouse placenta and suggest that caution should be made to avoid acute nanoparticle exposure during pregnancy [195].

In another study, Asharani and co-workers evaluated the toxicity of AgNPs in zebrafish models. The AgNPs were synthesized using starch and bovine serum albumin (BSA) as capping agents in order to examine their harmful effects and pattern of distribution in zebrafish embryos. The results revealed that in embryos treated with AgNPs, mortality increased in a dose-dependent manner, and hatching was delayed, while in developing embryos, the Ag^+^ ions and stabilizing agents did not reveal any notable toxic effects. These findings imply that AgNPs in embryos cause dose-dependent toxicity that inhibits normal development [196].

Furthermore, Teng et al. observed the impact of ZnO NP size on developmental toxicity to the foetus and placenta. After oral exposure, it was observed that smaller ZnO NPs (13 nm) were able to pass through the placental and intestinal barriers and reach the foetus where they induced developmental damage. Larger ZnO NPs (57 nm) and bulk ZnO particles, on the other hand, were unable to get past these barriers and have an effect [197]. Similarly, Yang et al. looked at the toxic effects of AuNP size and the gestational age of pregnant mice [198]. According to pharmacokinetic study results, NPs of 30 nm had a longer blood circulation period, while those of 4.5 nm were mostly eliminated in urine within 5 h. Different-sized AuNPs were administered intravenously to pregnant mice without causing any obvious harmful consequences, i.e., increased mortality, behavioural changes or decreased animal weight. However, the lungs of pregnant mice underwent moderate emphysema-like alterations after treatment with 30 nm AuNPs. These findings demonstrated that it was particle size, not gestational age, that determined the biodistribution patterns of AuNPs in pregnant mice. Moreover, organ-specific damage may be attributed to particle size [198,199]. There have been numerous studies aimed at assessing the toxicity associated with MNPs both in vitro and in animal models and are summarised in Table 4.

## 8. Conclusions and Prospects

Medicinal plants play a pivotal role in the biosynthesis of MNPs, and their reported use in theranostics is very promising. However, few plants have been investigated in this area. Studies on theranostics are limited and more research should be conducted to identify new chemical entities that are essential in capping and stabilizing MNPs. The potential role of theranostics still needs to be fully explored in diagnosing diseases, especially in cancer, malaria and cardiovascular diseases. Seemingly, the most-reported studies have focused on the therapeutic part and only a few studies have looked at the diagnosis of plant-derived MNPs. The effects of phytochemicals involved in the synthesis should also be looked into, as most studies report the use of plant extracts in green synthesis without mentioning the key phytochemicals involved.

Another concern is that the majority of published studies in this area are in vitro rather than in vivo research at the preclinical and clinical levels, making the current studies limited in substantiating their claims. In addition, other obstacles to the targeted delivery of encapsulated pharmaceuticals within nanoparticles include obtaining successful site-specific drug delivery and avoiding premature drug release. Until now, in vivo data have been limited to either medicinal or diagnostic research, rather than their combination, hence, signalling the need for developing the combination of the two. The other challenge is the use of plants in the biosynthesis of MNPs, as this may become a threat to medicinal plants that are prone to extinction. It is, therefore, imperative that future studies fully explore more sustainable extractions of medicinal plants, investigate in vivo studies, especially those that address the safety and efficacy of MNPs and research on the diagnostic use of MNPs.

## Figures and Tables

**Table 1:** Examples of phytochemicals that have been used in the biosynthesis of MNPs.

Plant	Compound	MW (g/mol)	MNP	Bioactivity	Reference
*Aspalathus linearis*	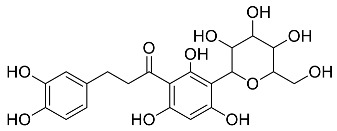 Aspalathin	452.13	AuNPs and RhNPs	Antimicrobial	[16,27]
*Caesalpinia spinosa*	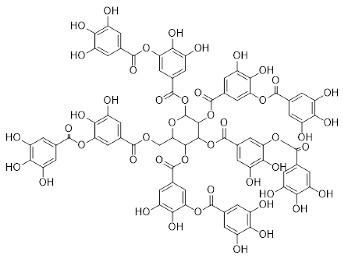 Tannic acid	1701.19	AuNPs	Antibacterial	[16,30,31]
*Centella asiatic*	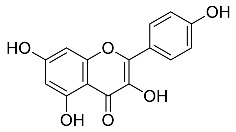 Kaempferol	2686.23	AuNPs	Anti-leishmanial	[16,32,33,34]
*Centella asiatic*	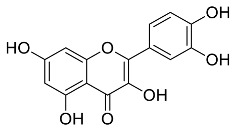 Quercetin	302.24	AgNPs	Antitumor, Antimicrobial	[34,35]
*Cinnamomum cassia*	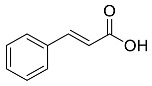 Cinnamic acid	148.15	AuNPs	Antimicrobial	[36,37]
*Cinnamomum zeylanicum*	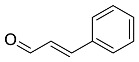 Cinnamaldehyde	132.16	AgNPs	Antimicrobial	[38,39]
*Cinnamomum zeylanicum* *verum*	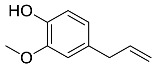 Eugenol	164.20	AgNPs	Antioxidant	[40,41]
*Citrus paradisi*	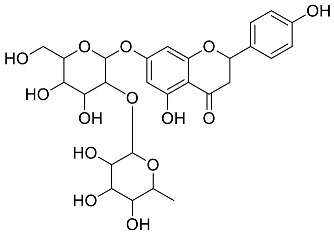 Naringin	580.54	AgNP	Antibacterial, Cytotoxic	[42,43]
*Citrus unshiu*	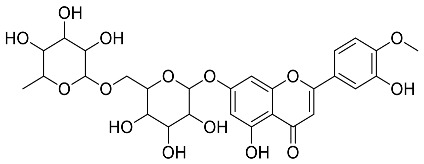 Narirutin	179.13	AuNPs	Antibacterial	[44,45]
*Coffea canephora*	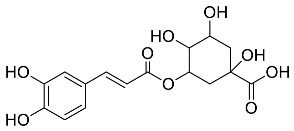 Chlorogenic acid	354.31	AgNPs	Antibacterial	[46,47]
*Curcuma longa*	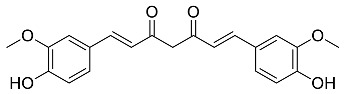 Curcumin	368.38	AgNPs	Antimicrobial	[48,49]
*Cyclopia intermedia*	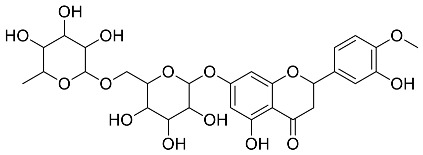 Hesperidin	610.19	AgNPs	Antibacterial, Cytotoxic	[42,50]
*Cynomorium coccineum*	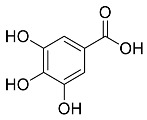 Gallic acid	170.12	AgNPs	Antimicrobial	[51,52]
*Eucalyptus globus*	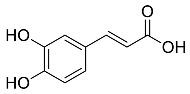 Caffeic acid	180.16	AgNPs	Anticancer	[53,54]
*Memecylon umbellatum*	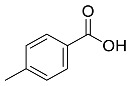 4-N-methylbenzoic acid	136.15	AgNPs	Antimicrobial, antioxidant, anticancertumor	[55]
*Mentha pulegium*	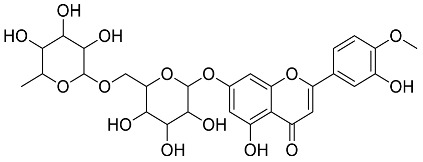 Diosmin	608.55	AgNPs	Antibacterial, Cytotoxic	[42,56]
*Myrica Esculenta*	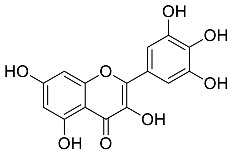 Myricetin	318.23	AuNPs	Anticancer	[57,58]
*Rubus idaeus*	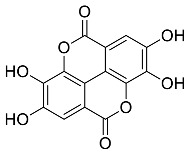 Ellagic acid	302.20	ZnNPs	Antiviral	[59,60]
*Stachys tuberifera*	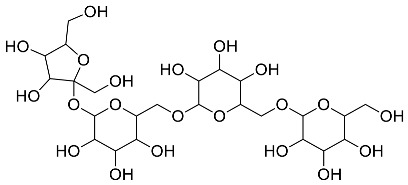 Stachyose	666.60	AgNPs	Antimicrobial	[61,62]
*Thymus vulgaris*	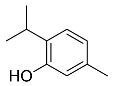 Thymol	150.22	AgNPs	Antimicrobial	[41,63]
*Vitis vinifera*	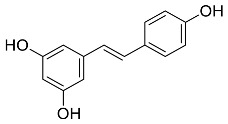 Resveratrol	228.25	AgNPs and AuNPs	Antibacterial	[64,65]

**Table 2:** A summary of the different characterisation techniques utilised for theranostics nanoparticles.

Characterization Technique	Physiochemical Parameter	Ref.
**Particle Size and Polydispersity Index**		
Atomic force microscopy (AFM)	This technique is used to determine the size and size distribution, shape, structure, dispersion and aggregation of the NPs.	[90,91]
Dynamic light scattering (DLS)	Essential in measuring crystallite size and for the distinction between the amorphous and the crystalline NPs. The dynamic light-scattering determines the size and quantification, while the transmission on the electron microscope is crucial in measuring the morphology and size of NPs.	[31,52,92,93]
Transmission scanning microscopy (TEM)	Images can be used to visualize the morphology of biosynthesized metallic NPs.	[64,94]
Scanning electron microscopy (SEM)	To determine the size as well as the morphology of NPs.	[49,95]
Fluorescence correlation spectroscopy (FCS)	A quantitative single-molecule technique that assesses the concentration and rate of diffusion of fluorophore-tagged molecules of all sizes in living cells and in vitro, as well as inside specific cellular compartments.	[91,96]
Scanning tunnelling microscopy (STM)	An analytical technique used to determine the surface composition through size and size distribution, shape, structure, dispersion and aggregation of the NPs.	[91,97]
Near-field scanning optical microscopy	A technique of microscopy for studying nanostructures that overcomes the far-field resolution barrier by taking advantage of evanescent wave properties.	[91,98]
**Zeta Potential**		
Electrophoretic Mobility (EM)	Used to determine the zeta potential, which is a measure or estimation of the colloidal stability.	[31,99,100]
**Chemical Composition and Surface Chemistry**		
Infrared spectroscopy (IR) Attenuated total reflection Fourier transform infrared (ATR–FTIR)	Provides data on the chemical composition (functional groups) of the structure of nanomaterials and conformation of the bioconjugates.	[91,97,101]
Mass spectroscopy (MS)	Used to determine the mass-to-charge ratio of molecules in a sample.	[91,102]
X-ray Photoelectron Spectroscopy (XPS)	A powerful quantitative technique often used to elucidate the electronic structure, elemental composition and oxidation states of elements in a nanomaterial.	[103,104]
Nuclear magnetic resonance (NMR)	Used to determine the size through indirect analysis, structure, composition, purity and conformational change.	[91,105]
Raman scattering (RS) Surface-enhanced Raman (SERS), Tip-enhanced Raman spectroscopy (TERS)	Primarily, it identifies the NPs’ structural, chemical and electrical properties. It can also be used to calculate the protein-metallic nanoparticle conjugate’s hydrodynamic size and size distribution.	[91,100]
**Crystal Habit**		
Thermal gravimetric analysis (TGA)	Used to evaluate the weight shift that takes place as a sample, it is heated at a constant rate in order to measure the percentage of volatile components and the thermal stability of a material. It can also be used to determine the changes in polymorph by noting whether a sample is a hydrate or solvate.	[89,106]
Differential Scanning Calorimetry	Utilises the difference in the amount of heat required to increase the temperature of a sample and a reference. It can determine whether the sample is amorphous or crystalline as well as determining if a polymorphic change has occurred.	[89,107]
X-ray diffraction (XRD)	A technique to determine the size, shape and structure for nano materials or crystals.	[91,108]
**Optical Properties**		
Ultraviolet, visible, near infrared (UV–vis–NIR) spectroscopy	Predominantly used in determining the surface plasmon resonance (SPR) of MNPs. Reported studies have determined metals such as gold to be identified at wavelengths of 520–560 nm.	[46,87,94]

**Table 3:** Some of the plant species that have been used to synthesize metallic nanoparticles through green technology.

Plant	MNPs	Morphology	Application	Reference
*Acalypha indica*	AgNPs	Spherical	Antimicrobial	[145]
*Acalypha indica*	AgNPs AuNPs	Spherical	Anticancer	[146]
*Alternanthera sessilis*	AgNPs	Spherical	Antimicrobial	[147]
*Anisomeles indica*	AgNPs	Spherical	Antimalarial	[19]
*Artemisia annua*	ZnONPs	Spherical	Anticancer	[148]
*Carica papaya*	AgNPs	Spherical	Anticancer	[149]
*Cassia alata*	ZnONPs	Spherical	Antimicrobial	[150]
*Catharanthus roseus*	AgNPs	Spherical	Antimicrobial	[151]
*Crataegus ambigua*	AgNPs	Spherical	Antimalarial, antimicrobial	[144]
*Cyclopia intermedia*	AuNPs	Spherical and triangular	Anticancer	[144]
*Echinochloa frumentacea*	ZnONPs	Hexagonal	Antimicrobial	[152]
*Euphorbia hirta*	ZnONPs	Spherical	Antimicrobial	[153]
*Leucas aspera* and *Hyptis suaveolens*	AgNPs	Spherical, hexagonal, triangular, and polyhedral	Antimalarial	[154]
*Mangifera indica*	AuNPs	Spherical	Anticancer	[155]
*Melia azedarach*	ZnONPs	Spherical and hexagonal	Antimicrobial	[156]
*Mirabilis jalapa*	AuNPs	Spherical	Antimicrobial	[4,157]
*Morus nigra*	ZnONPs	Spherical	Anticancer	[158]
*Nepenthes khasiana*	AuNPs	Triangular and spherical	Antimicrobial	[159]
*Pinus thunbergia*	AgNPs	Triangular and hexagonal	Antimicrobial	[160]
*Plumbago auriculata*	AgNPs	Spherical and oblong	Antimicrobial	[161]
*Pteridium aquilinum*	AgNPs	Spherical	Antimalarial	[162]
*Rhizoma paridis*	ZnONPs	Spherical	Anticancer	[163]
*Salvia* *africana-lutea* and *Sutherlandia frutescens*	AgNPs AuNPs	Spherical and Polygon	Antimicrobial, anticancer	[164]
*Salvia officinalis*	AgNPs	Spherical	Antimalarial	[143]
*Vaccinium arctostaphylos*	ZnONPs	Spindle	Antidiabetic, antimicrobial	[165]
*Zingiber officinale* and *Allium sativum*	NiONPs	Spherical	Antimicrobial	[166]

**Table 4:** Summary of MNPs that have been assessed for their biological safety using in vitro and in vivo models.

MNPs	Size (nm)	Model	Toxic Effect(s)	Ref.
AgNPs	30–50	Rat hepatic stellate cells	Proliferative and apoptotic effect	[200]
AgNPs	70	Rat	Tissue damages, bloodshed, cell necrosis	[201]
AgNPs	15, 100	Rat liver cells BRL 3A (ATCC, CRL-1442)	Decreased mitochondrial function	[202]
AgNPs	35–100	Mice	Alteration of neonatal kidney	[203]
AgNPs	20	Rat	Induce oxidative stress and apoptosis in the liver	[204]
AgNPs	10, 40, 100	Rat	Hepatobiliary toxicity	[205]
AuNPs	40, 100	Mice	Changes miRNA expression in foetus	[206]
AuNPs	5, 10, 30, 60	Mice	Elevation of liver enzymes, accumulation in the liver and spleen	[207]
AuNPs	20	Human lung adenocarcinoma cells (A549 cells)	Causes cell damage	[208]
AuNPs	4.4–36.1	Mice	Causes liver and kidney damage	[209]
AuNPs	20	Rat	Spleen atrophy	[210]
TiO_2_NPs	<25	Rat	Teratogenic (impairs foetal skeletal formation, causes weight loss, liver and kidney degeneration)	[211]
ZnONPs	34–40	Rat	Affects hepatic and renal performance, cumulative toxicity	[212]
ZnONPs	20,120	Mice	Weight loss, liver damage, accumulation of Zn in the liver and kidney	[213]

## Data Availability

Not applicable.

## References

[1] Rafael F., Coutinho A.M., Costa L.B., Barbosa F.G., Queiroz M.A., Cerri G.G. (2020). Theranostics in Nuclear Medicine: Emerging and Re-Emerging Inte- Grated Imaging and Therapies in the Era of Precision Oncology. Radiographics.

[2] Filippov A., Bonjoc K.C., Chea J., Bowles N., Poku E. (2020). Role of Theranostics in Thoracic Oncology. J. Thorac. Dis..

[3] Bhattacharya S., Alkharfy K.M., Janardhanan R., Mukhopadhyay D. (2012). Nanomedicine: Pharmacological Perspectives. Nanotechnol. Rev..

[4] Mikhailova E.O. (2021). Gold Nanoparticles: Biosynthesis and Potential of Biomedical Application. J. Funct. Biomater..

[5] Liu L., Kshirsagar P.G., Gautam S.K., Gulati M., Wafa E.I., Christiansen J.C., White B.M., Mallapragada S.K., Wannemuehler M.J., Kumar S. (2022). Nanocarriers for Pancreatic Cancer Imaging, Treatments, and Immunotherapies. Theranostics.

[6] Krzyszczyk P., Acevedo A., Davidoff E.J., Timmins L.M., Marrero-Berrios I., Patel M., White C., Lowe C., Sherba J.J., Hartmanshenn C. (2018). The Growing Role of Precision and Personalized Medicine for Cancer Treatment. Technology.

[7] WHO Catalysing Ancient Wisdom and Modern Science for the Health of People and the Planet. https://www.who.int/initiatives/who-global-centre-for-traditional-medicine.

[8] Woodley C.M., Amado P.S.M., Cristiano M.L.S., O’Neill P.M. (2021). Artemisinin Inspired Synthetic Endoperoxide Drug Candidates: Design, Synthesis, and Mechanism of Action Studies. Med. Res. Rev..

[9] Whayne T.F. (2018). Clinical Use of Digitalis: A State of the Art Review. Am. J. Cardiovasc. Drugs.

[10] Sharma A., Kontodimas K., Bosmann M. (2021). Nanomedicine: A Diagnostic and Therapeutic Approach to COVID-19. Front. Med..

[11] Shah M., Fawcett D., Sharma S., Tripathy S.K., Poinern G.E.J. (2015). Green Synthesis of Metallic Nanoparticles via Biological Entities. Materials.

[12] Gandhi P.R., Jayaseelan C., Kamaraj C., Rajasree S.R.R., Regina Mary R. (2018). In Vitro Antimalarial Activity of Synthesized TiO2 Nanoparticles Using Momordica Charantia Leaf Extract against Plasmodium Falciparum. J. Appl. Biomed..

[13] Bharadwaj K.K., Rabha B., Pati S., Sarkar T., Choudhury B.K., Barman A., Bhattacharjya D., Srivastava A., Baishya D., Edinur H.A. (2021). Green Synthesis of Gold Nanoparticles Using Plant Extracts as Beneficial Prospect for Cancer Theranostics. Molecules.

[14] Yuan P., Ding X., Yang Y.Y., Xu Q.H. (2018). Metal Nanoparticles for Diagnosis and Therapy of Bacterial Infection. Adv. Healthc. Mater..

[15] Oshadie G., Silva D., Abeysundara A.T., Minoli M., Aponso W. (2017). Extraction Methods, Qualitative and Quantitative Techniques for Screening of Phytochemicals from Plants. Am. J. Essent. Oils Nat. Prod..

[16] Aboyewa J.A., Sibuyi N.R.S., Meyer M., Oguntibeju O.O. (2021). Green Synthesis of Metallic Nanoparticles Using Some Selected Medicinal Plants from Southern Africa and Their Biological Applications. Plants.

[17] Iravani S., Korbekandi H., Mirmohammadi S.V., Zolfaghari B. (2014). Synthesis of Silver Nanoparticles: Chemical, Physical and Biological Methods. Res. Pharm. Sci..

[18] Kharissova O.V., Kharisov B.I., González C.M.O., Méndez Y.P., López I. (2019). Greener Synthesis of Chemical Compounds and Materials. R. Soc. Open Sci..

[19] Govindarajan M., Rajeswary M., Veerakumar K., Muthukumaran U., Hoti S.L., Benelli G. (2016). Green Synthesis and Characterization of Silver Nanoparticles Fabricated Using *Anisomeles Indica*: Mosquitocidal Potential against Malaria, Dengue and Japanese Encephalitis Vectors. Exp. Parasitol..

[20] Verma A., Gautam S., Bansal K., Prabhakar N., Rosenholm J. (2019). Green Nanotechnology: Advancement in Phytoformulation Research. Medicines.

[21] Ovais M., Khalil A.T., Raza A., Islam N.U., Ayaz M., Saravanan M., Ali M., Ahmad I., Shahid M., Shinwari Z.K. (2018). Multifunctional Theranostic Applications of Biocompatible Green-Synthesized Colloidal Nanoparticles. Appl. Microbiol. Biotechnol..

[22] Kuppusamy P., Yusoff M.M., Maniam G.P., Govindan N. (2016). Biosynthesis of Metallic Nanoparticles Using Plant Derivatives and Their New Avenues in Pharmacological Applications—An Updated Report. Saudi Pharm. J..

[23] Parveen K., Banse V., Ledwani L. (2016). Green Synthesis of Nanoparticles: Their Advantages and Disadvantages. AIP Conf. Proc..

[24] Ovais M., Khalil A.T., Islam N.U., Ahmad I., Ayaz M., Saravanan M., Shinwari Z.K., Mukherjee S. (2018). Role of Plant Phytochemicals and Microbial Enzymes in Biosynthesis of Metallic Nanoparticles. Appl. Microbiol. Biotechnol..

[25] Jamkhande P.G., Ghule N.W., Bamer A.H., Kalaskar M.G. (2019). Metal Nanoparticles Synthesis: An Overview on Methods of Preparation, Advantages and Disadvantages, and Applications. J. Drug Deliv. Sci. Technol..

[26] Nazli A., Baig M.W., Zia M., Ali M., Shinwari Z.K., Ul Haq I. (2018). Plant-Based Metallic Nanoparticles as Potential Theranostics Agents: Bioinspired Tool for Imaging and Treatment. IET Nanobiotechnology.

[27] Johnson R., De Beer D., Dludla P.V., Ferreira D., Muller C.J.F., Joubert E. (2018). Aspalathin from Rooibos (*Aspalathus Linearis*): A Bioactive C -Glucosyl Dihydrochalcone with Potential to Target the Metabolic Syndrome. Planta Med..

[28] Rai M., Yadav A. (2013). Plants as Potential Synthesiser of Precious Metal Nanoparticles: Progress and Prospects. IET Nanobiotechnology.

[29] Ponnuchamy K., Jacob J.A. (2016). Metal Nanoparticles from Marine Seaweeds—A Review. Nanotechnol. Rev..

[30] Romero N., Fernández A., Robert P. (2012). A Polyphenol Extract of Tara Pods (*Caesalpinia Spinosa*) as a Potential Antioxidant in Oils. Eur. J. Lipid Sci. Technol..

[31] Alshammari A., Köckritz A., Narayana Kalevaru V., Bagabas A., Martin A. (2012). Influence of Single Use and Combination of Reductants on the Size, Morphology and Growth Steps of Gold Nanoparticles in Colloidal Mixture. Open J. Phys. Chem..

[32] Jeganathan B., Punyasiri P.A.N., Kottawa-Arachchi J.D., Ranatunga M.A.B., Abeysinghe I.S.B., Gunasekare M.T.K., Bandara B.M.R. (2016). Genetic Variation of Flavonols Quercetin, Myricetin, and Kaempferol in the Sri Lankan Tea (*Camellia Sinensis* L.) and Their Health-Promoting Aspects. Int. J. Food Sci..

[33] Halder A., Das S., Bera T., Mukherjee A. (2017). Rapid Synthesis for Monodispersed Gold Nanoparticles in Kaempferol and Anti-Leishmanial Efficacy against Wild and Drug Resistant Strains. RSC Adv..

[34] Joshi C., Savai J., Varghese A., Pandita N. (2012). Development and Validation of HPTLC Method for Simultaneous Determination of Quercetin and Kaempferol in Leaves of Two Chemotypes of *Centella asiatica*. J. Planar Chromatogr.—Mod. TLC.

[35] Mittal A.K., Kumar S., Banerjee U.C. (2014). Quercetin and Gallic Acid Mediated Synthesis of Bimetallic (Silver and Selenium) Nanoparticles and Their Antitumor and Antimicrobial Potential. J. Colloid Interface Sci..

[36] Lakshmi B.S., Sujatha S., Anand S., Sangeetha K.N., Narayanan R.B., Katiyar C., Kanaujia A., Duggar R., Singh Y., Srinivas K. (2009). Cinnamic Acid, from the Bark of *Cinnamomum Cassia*, Regulates Glucose Transport via Activation of GLUT4 on L6 Myotubes in a Phosphatidylinositol 3-Kinase-Independent Manner. J. Diabetes.

[37] Anwar A., Siddiqui R., Shah M.R., Khan N.A. (2018). Gold nanoparticle-conjugated cinnamic acid exhibits antiacanthamoebic and antibacterial properties. Antimicrob. Agents Chemother..

[38] Al-Bayati F.A., Mohammed M.J. (2009). Isolation, Identification, and Purification of Cinnamaldehyde from *Cinnamomum Zeylanicum* Bark Oil. An Antibacterial Study. Pharm. Biol..

[39] Ramasamy M., Lee J., Lee J. (2017). IJN-132784-Development-of-Gold-Nanoparticles-Coated-with-Silica-Contain. Int. J. Nanomedicine.

[40] Ranasinghe L., Jayawardena B., Abeywickrama K. (2002). Fungicidal Activity of Essential Oils of *Cinnamomum Zeylanicum* (L.) and *Syzygium Aromaticum* (L.) Merr et L.M.Perry against Crown Rot and Anthracnose Pathogens Isolated from Banana. Lett. Appl. Microbiol..

[41] Abed M.S., Abed A.S., Othman F.M. (2019). Green Synthesis of Silver Nanoparticles from Natural Compounds: Glucose, Eugenol and Thymol. J. Adv. Res. Fluid Mech. Therm. Sci..

[42] Sahu N., Soni D., Chandrashekhar B., Satpute D.B., Saravanadevi S., Sarangi B.K., Pandey R.A. (2016). Synthesis of Silver Nanoparticles Using Flavonoids: Hesperidin, Naringin and Diosmin, and Their Antibacterial Effects and Cytotoxicity. Int. Nano Lett..

[43] Giannuzzo A.N., Boggetti H.J., Nazareno M.A., Mishima H.T. (2003). Supercritical Fluid Extraction of Naringin from the Peel of *Citrus paradisi*. Phytochem. Anal..

[44] Yuan C.G., Huo C., Gui B., Cao W.P. (2017). Green Synthesis of Gold Nanoparticles Using *Citrus Maxima* Peel Extract and Their Catalytic/Antibacterial Activities. IET Nanobiotechnol..

[45] Park H.Y., Ha S.K., Eom H., Choi I. (2013). Narirutin Fraction from Citrus Peels Attenuates Alcoholic Liver Disease in Mice. Food Chem. Toxicol..

[46] Noh H.J., Kim H.S., Jun S.H., Kang Y.H., Cho S., Park Y. (2013). Biogenic Silver Nanoparticles with Chlorogenic Acid as a Bioreducing Agent. J. Nanosci. Nanotechnol..

[47] Mahesh V., Million-Rousseau R., Ullmann P., Chabrillange N., Bustamante J., Mondolot L., Morant M., Noirot M., Hamon S., De Kochko A. (2007). Functional Characterization of Two P-Coumaroyl Ester 3′-Hydroxylase Genes from Coffee Tree: Evidence of a Candidate for Chlorogenic Acid Biosynthesis. Plant Mol. Biol..

[48] Revathy S., Elumalai S., Benny M., Antony B. (2011). Isolation, Purification and Identification of Curcuminoids from Turmeric (*Curcuma Longa* L.) by Column Chromatography. J. Exp. Sci..

[49] Jaiswal S., Mishra P. (2018). Antimicrobial and Antibiofilm Activity of Curcumin-Silver Nanoparticles with Improved Stability and Selective Toxicity to Bacteria over Mammalian Cells. Med. Microbiol. Immunol..

[50] Bartoszewski R., Hering A., Marszałł M., Hajduk J.S., Bartoszewska S., Kapoor N., Kochan K., Ochocka R. (2014). Mangiferin Has an Additive Effect on the Apoptotic Properties of Hesperidin in Cyclopia Sp. Tea Extracts. PLoS ONE.

[51] Zucca P., Rosa A., Tuberoso C.I.G., Piras A., Rinaldi A.C., Sanjust E., Dessì M.A., Rescigno A. (2013). Evaluation of Antioxidant Potential of “Maltese Mushroom” (*Cynomorium coccineum*) by Means of Multiple Chemical and Biological Assays. Nutrients.

[52] Li D., Liu Z., Yuan Y., Liu Y., Niu F. (2015). Green Synthesis of Gallic Acid-Coated Silver Nanoparticles with High Antimicrobial Activity and Low Cytotoxicity to Normal Cells. Process Biochem..

[53] Pan M., Lei Q., Zang N., Zhang H. (2019). A Strategy Based on GC-MS/MS, UPLC-MS/MS and Virtual Molecular Docking for Analysis and Prediction of Bioactive Compounds in *Eucalyptus Globulus* Leaves. Int. J. Mol. Sci..

[54] Guo D., Dou D., Ge L., Huang Z., Wang L., Gu N. (2015). A Caffeic Acid Mediated Facile Synthesis of Silver Nanoparticles with Powerful Anti-Cancer Activity. Colloids Surf. B Biointerfaces.

[55] AlSalhi M.S., Elangovan K., Ranjitsingh A.J.A., Murali P., Devanesan S. (2019). Synthesis of Silver Nanoparticles Using Plant Derived 4-N-Methyl Benzoic Acid and Evaluation of Antimicrobial, Antioxidant and Antitumor Activity. Saudi J. Biol. Sci..

[56] Aires A., Marrinhas E., Carvalho R., Dias C., Saavedra M.J. (2016). Phytochemical Composition and Antibacterial Activity of Hydroalcoholic Extracts of Pterospartum Tridentatum and Mentha Pulegium against *Staphylococcus aureus* Isolates. Biomed Res. Int..

[57] Mohan U.P., Sriram B., Panneerselvam T., Devaraj S., MubarakAli D., Parasuraman P., Palanisamy P., Premanand A., Arunachalam S., Kunjiappan S. (2020). Utilization of Plant-Derived Myricetin Molecule Coupled with Ultrasound for the Synthesis of Gold Nanoparticles against Breast Cancer. Naunyn. Schmiedebergs. Arch. Pharmacol..

[58] Patel K.G., Patel V.G., Patel K.V., Gandhi T.R. (2010). Validated HPTLC Method for Quantification of Myricetin in the Stem Bark of Myrica Esculenta Buch. Ham. Ex D. Don, Myricaceae. J. Planar Chromatogr.—Mod. TLC.

[59] Abouaitah K., Allayh A.K., Wojnarowicz J., Shaker Y.M., Swiderska-Sroda A., Lojkowski W. (2021). Nanoformulation Composed of Ellagic Acid and Functionalized Zinc Oxide Nanoparticles Inactivates Dna and Rna Viruses. Pharmaceutics.

[60] Mullen W., Yokota T., Lean M.E.J., Crozier A. (2003). Analysis of Ellagitannins and Conjugates of Ellagic Acid and Quercetin in Raspberry Fruits by LC-MSn. Phytochemistry.

[61] Raju D., Muchintala D., Reddy M., Rao B. (2018). Synthesis and Characterization of Oligosaccharide Based Silver Nanoparticles and Its Assessment as an Antimicrobial Agent. Adv. Sci. Eng. Med..

[62] Wild G.M., French D. (1952). The galactan series of oligosaccharides. Proc. Iowa Acad. Sci. Galactan Ser. Oligosacch..

[63] Zeng Q., Che Y., Zhang Y., Chen M., Guo Q., Zhang W. (2020). Thymol Isolated from *Thymus Vulgaris* L. Inhibits Colorectal Cancer Cell Growth and Metastasis by Suppressing the Wnt/β-Catenin Pathway. Drug Des. Devel. Ther..

[64] Park S., Cha S.H., Cho I., Park S., Park Y., Cho S., Park Y. (2016). Antibacterial Nanocarriers of Resveratrol with Gold and Silver Nanoparticles. Mater. Sci. Eng. C.

[65] Mattivi F., Vrhovsek U., Malacarne G., Masuero D., Zulini L., Stefanini M., Mose C., Velasco R., Guella G. (2011). Profiling of Resveratrol Oligomers, Important Stress Metabolites, Accumulating in the Leaves of Hybrid *Vitis vinifera* (Merzling × Teroldego) Genotypes Infected with *Plasmopara viticola*. J. Agric. Food Chem..

[66] Chinnappan R.S., Kandasamy K., Sekar A. (2015). A Review on Marine Based Nanoparticles and Their Potential Applications. Afr. J. Biotechnol..

[67] Ghosh V. (2020). Marine Bioresources as Potential Source for Synthesis of Nanoparticles. Encycl. Mar. Biotechnol..

[68] Javed R., Zia M., Naz S., Aisida S.O., ul Ain N., Ao Q. (2020). Role of Capping Agents in the Application of Nanoparticles in Biomedicine and Environmental Remediation: Recent Trends and Future Prospects. J. Nanobiotechnol..

[69] Ahmad T., Bustam M.A., Irfan M., Moniruzzaman M., Asghar H.M.A., Bhattacharjee S. (2019). Mechanistic Investigation of Phytochemicals Involved in Green Synthesis of Gold Nanoparticles Using Aqueous Elaeis Guineensis Leaves Extract: Role of Phenolic Compounds and Flavonoids. Biotechnol. Appl. Biochem..

[70] Elbagory A.M., Cupido C.N., Meyer M., Hussein A.A. (2016). Large Scale Screening of Southern African Plant Extracts for the Green Synthesis of Gold Nanoparticles Using Microtitre-Plate Method. Molecules.

[71] Khairunnisa S., Wonoputri V., Samadhi T.W. (2021). Effective Deagglomeration in Biosynthesized Nanoparticles: A Mini Review. IOP Conf. Ser. Mater. Sci. Eng..

[72] Dumur F., Guerlin A., Dumas E., Bertin D., Gigmes D., Mayer C.R. (2011). Controlled Spontaneous Generation of Gold Nanoparticles Assisted by Dual Reducing and Capping Agents. Gold Bull..

[73] Niu Z., Li Y. (2014). Removal and Utilization of Capping Agents in Nanocatalysis. Chem. Mater..

[74] Ali S., Sharma A.S., Ahmad W., Zareef M., Hassan M.M., Viswadevarayalu A., Jiao T., Li H., Chen Q. (2021). Noble Metals Based Bimetallic and Trimetallic Nanoparticles: Controlled Synthesis, Antimicrobial and Anticancer Applications. Crit. Rev. Anal. Chem..

[75] Ribeiro A.I., Dias A.M., Zille A. (2022). Synergistic Effects between Metal Nanoparticles and Commercial Antimicrobial Agents: A Review. ACS Appl. Nano Mater..

[76] Melkamu W.W., Bitew L.T. (2021). Green Synthesis of Silver Nanoparticles Using *Hagenia abyssinica* (Bruce) J.F. Gmel Plant Leaf Extract and Their Antibacterial and Anti-Oxidant Activities. Heliyon.

[77] Ray P.C., Yu H., Fu P.P. (2009). Toxicity and environmental risks of nanomaterials: Challenges and future needs. J. Environ. Sci. Health Part C.

[78] Adeyemi J.O., Oriola A.O., Onwudiwe D.C., Oyedeji A.O. (2022). Plant Extracts Mediated Metal-Based Nanoparticles: Synthesis and Biological Applications. Biomolecules.

[79] Xu L., Wang Y.-Y., Huang J., Chen C.-Y., Wang Z.-X., Xie H. (2020). Silver Nanoparticles: Synthesis, Medical Applications and Biosafety. Theranostics.

[80] Habeeb Rahuman H.B., Dhandapani R., Narayanan S., Palanivel V., Paramasivam R., Subbarayalu R., Thangavelu S., Muthupandian S. (2022). Medicinal Plants Mediated the Green Synthesis of Silver Nanoparticles and Their Biomedical Applications. IET Nanobiotechnol..

[81] El Shafey A.M. (2020). Green Synthesis of Metal and Metal Oxide Nanoparticles from Plant Leaf Extracts and Their Applications: A Review. Green Process. Synth..

[82] Raj S., Trivedi R., Soni V. (2021). Biogenic Synthesis of Silver Nanoparticles, Characterization and Their Applications—A Review. Surfaces.

[83] Iqbal P., Preece J.A., Mendes P.M. (2012). Nanotechnology: The “Top-Down” and “Bottom-Up” Approaches. Supramol. Chem..

[84] Tsuzuki T. (2009). Commercial Scale Production of Inorganic Nanoparticles. Int. J. Nanotechnol..

[85] Aliofkhazraei M. (2011). Synthesis, Processing and Application of Nanostructured Coatings. Eng. Mater..

[86] Zhang M., Yang J., Cai Z., Feng Y., Wang Y., Zhang D., Pan X. (2019). Detection of Engineered Nanoparticles in Aquatic Environments: Current Status and Challenges in Enrichment, Separation, and Analysis. Environ. Sci. Nano.

[87] Jabir M.S., Taha A.A., Sahib U.I. (2018). Linalool Loaded on Glutathione-Modified Gold Nanoparticles: A Drug Delivery System for a Successful Antimicrobial Therapy. Artif. Cells Nanomed. Biotechnol..

[88] Kumar A., Chaudhary R.K., Singh R., Singh S.P., Wang S.Y., Hoe Z.Y., Pan C.T., Shiue Y.L., Wei D.Q., Kaushik A.C. (2020). Nanotheranostic Applications for Detection and Targeting Neurodegenerative Diseases. Front. Neurosci..

[89] Witika B.A., Aucamp M., Mweetwa L.L., Makoni P.A. (2021). Application of Fundamental Techniques for Physicochemical Characterizations to Understand Post-Formulation Performance of Pharmaceutical Nanocrystalline Materials. Crystals.

[90] Brar S.K., Verma M. (2011). Measurement of Nanoparticles by Light-Scattering Techniques. TrAC Trends Anal. Chem..

[91] Lin P.-C., Lin S., Wang P.C., Sridhar R. (2014). Techniques for Physicochemical Characterization of Nanomaterials. Biotechnol. Adv..

[92] Avitabile E., Senes N., D’Avino C., Tsamesidis I., Pinna A., Medici S., Pantaleo A. (2020). The Potential Antimalarial Efficacy of Hemocompatible Silver Nanoparticles from *Artemisia* Species against *P. Falciparum* Parasite. PLoS ONE.

[93] Bhattacharjee S. (2016). DLS and Zeta Potential—What They Are and What They Are Not?. J. Control. Release.

[94] Patra B., Gautam R., Priyadarsini E., Rajamani P., Pradhan S.N., Saravanan M., Meena R. (2020). Piper Betle: Augmented Synthesis of Gold Nanoparticles and Its In-Vitro Cytotoxicity Assessment on HeLa and HEK293 Cells. J. Clust. Sci..

[95] Baalousha M., Ju-Nam Y., Cole P.A., Gaiser B., Fernandes T.F., Hriljac J.A., Jepson M.A., Stone V., Tyler C.R., Lead J.R. (2012). Characterization of Cerium Oxide Nanoparticles-Part 1: Size Measurements. Environ. Toxicol. Chem..

[96] Knox S.L., Steinauer A., Alpha-Cobb G., Trexler A., Rhoades E., Schepartz A. (2020). Chapter Twenty-One—Quantification of Protein Delivery in Live Cells Using Fluorescence Correlation Spectroscopy. Chemical Tools for Imaging, Manipulating, and Tracking Biological Systems: Diverse Chemical, Optical and Bioorthogonal Methods.

[97] Patil R.B., Chougale A.D. (2021). Analytical Methods for the Identification and Characterization of Silver Nanoparticles: A Brief Review. Mater. Today Proc..

[98] Hartschuh A., Bhushan B. (2016). Scanning Near-Field Optical Microscopy BT—Encyclopedia of Nanotechnology.

[99] Kaszuba M., McKnight D., Connah M.T., McNeil-Watson F.K., Nobbmann U. (2008). Measuring Sub Nanometre Sizes Using Dynamic Light Scattering. J. Nanopart. Res..

[100] Li Y., Lee J.S. (2020). Insights into Characterization Methods and Biomedical Applications of Nanoparticle-Protein Corona. Materials.

[101] Sapsford K.E., Tyner K.M., Dair B.J., Deschamps R., Medintz I.L. (2011). Analyzing Nanomaterial Bioconjugates: A Review of Current and Emerging Purification and Characterization Techniques. Anal. Chem..

[102] Mernie E.G., Chen Y. (2022). Nanoprobe-Based Mass Spectrometry and Fourier Transform Infrared Spectroscopy for Rapid Phospholipid Profiling. J. Chin. Chem. Soc..

[103] Mourdikoudis S., Pallares R.M., Thanh N.T.K. (2018). Characterization Techniques for Nanoparticles: Comparison and Complementarity upon Studying Nanoparticle Properties. Nanoscale.

[104] Sarma D.D., Santra P.K., Mukherjee S., Nag A. (2013). X-Ray Photoelectron Spectroscopy: A Unique Tool to Determine the Internal Heterostructure of Nanoparticles. Chem. Mater..

[105] Perera Y.R., Hill R.A., Fitzkee N.C. (2019). Protein Interactions with Nanoparticle Surfaces: Highlighting Solution NMR Techniques. Isr. J. Chem..

[106] Thomas L.C., Schmidt S.J., Nielsen S.S. (2017). Thermal Analysis BT—Food Analysis.

[107] Witika B.A., Smith V.J., Walker R.B. (2020). A Comparative Study of the Effect of Different Stabilizers on the Critical Quality Attributes of Self-Assembling Nano Co-Crystals. Pharmaceutics.

[108] Giannini C., Ladisa M., Altamura D., Siliqi D., Sibillano T., De Caro L. (2016). X-Ray Diffraction: A Powerful Technique for the Multiple-Length-Scale Structural Analysis of Nanomaterials. Crystals.

[109] Jain A., Jain P., Soni P., Tiwari A., Prasad S. (2021). Design and Characterization of Silver Nanoparticles of Different Species of Curcuma in the Treatment of Cancer Using Human Colon Cancer Cell Line (HT—29). J. Gastrointest. Cancer.

[110] Kristanc L., Kreft S. (2016). European Medicinal and Edible Plants Associated with Subacute and Chronic Toxicity Part I: Plants with Carcinogenic, Teratogenic and Endocrine-Disrupting Effects. Food Chem. Toxicol..

[111] Novak M., Žegura B., Modic B., Heath E., Filipič M. (2017). Cytotoxicity and Genotoxicity of Anticancer Drug Residues and Their Mixtures in Experimental Model with Zebrafish Liver Cells. Sci. Total Environ..

[112] Weber G.F. (2014). DNA Damaging Drugs. Molecular Therapies of Cancer.

[113] Greenwell M., Rahman P.K.S.M. (2015). Medicinal Plants: Their Use in Anticancer Treatment. Int. J. Pharm. Sci. Res..

[114] Gao Y., Shang Q., Li W., Guo W., Stojadinovic A., Mannion C., Man Y.-G., Chen T. (2020). Antibiotics for Cancer Treatment: A Double-Edged Sword. J. Cancer.

[115] Hailan W.A., Al-Anazi K.M., Farah M.A., Ali M.A., Al-Kawmani A.A., Abou-Tarboush F.M. (2022). Reactive Oxygen Species-Mediated Cytotoxicity in Liver Carcinoma Cells Induced by Silver Nanoparticles Biosynthesized Using Schinus Molle Extract. Nanomaterials.

[116] Tailor G., Lawal A.M. (2021). Phytochemical Screening; Green Synthesis, Characterization and Biological Significance of Lead Oxide Nanoparticles from *Eucalyptus globulus* Labill. (Leaves). Nanotechnol. Environ. Eng..

[117] Ramamurthy C.H., Padma M., Mareeswaran R., Suyavaran A., Kumar M.S., Premkumar K., Thirunavukkarasu C. (2013). The Extra Cellular Synthesis of Gold and Silver Nanoparticles and Their Free Radical Scavenging and Antibacterial Properties. Colloids Surf. B Biointerfaces.

[118] Slavin Y.N., Asnis J., Häfeli U.O., Bach H. (2017). Metal Nanoparticles: Understanding the Mechanisms behind Antibacterial Activity. J. Nanobiotechnol..

[119] Antimicrobial Resistance. https://www.who.int/news-room/fact-sheets/detail/antimicrobial-resistance.

[120] Tong S.Y.C., Davis J.S., Eichenberger E., Holland T.L., Fowler Jr V.G. (2015). *Staphylococcus aureus* Infections: Epidemiology, Pathophysiology, Clinical Manifestations, and Management. Clin. Microbiol. Rev..

[121] Celebioglu A., Topuz F., Yildiz Z.I., Uyar T. (2019). One-Step Green Synthesis of Antibacterial Silver Nanoparticles Embedded in Electrospun Cyclodextrin Nanofibers. Carbohydr. Polym..

[122] Attallah N.G.M., Elekhnawy E., Negm W.A., Hussein I.A., Mokhtar F.A., Al-Fakhrany O.M. (2022). In Vivo and in Vitro Antimicrobial Activity of Biogenic Silver Nanoparticles against *Staphylococcus aureus* Clinical Isolates. Pharmaceuticals.

[123] World Health Organization Cardiovascular Diseases (cvds). https://www.who.int/news-room/fact-sheets/detail/cardiovascular-diseases-.

[124] Aronson D., Edelman E.R. (2010). Revascularization for Coronary Artery Disease in Diabetes Mellitus: Angioplasty, Stents and Coronary Artery Bypass Grafting. Rev. Endocr. Metab. Disord..

[125] Bardage C., Isacson D.G.L. (2000). Self-Reported Side-Effects of Antihypertensive Drugs: An Epidemiological Study on Prevalence and Impact on Health-State Utility. Blood Press..

[126] Olivier T.T., Moïse F., Jackson S.A., Francis N.T. (2016). A Review on Traditional Uses, Phytochemical and Pharmacological Profiles, Spiritual and Economic Values, and Toxicity of *Dacryodes Edulis* (G. Don) H.J. Lam. J. Drug Deliv. Ther..

[127] Stendahl J.C., Sinusas A.J. (2015). Nanoparticles for Cardiovascular Imaging and Therapeutic Delivery, Part 1: Compositions and Features. J. Nucl. Med..

[128] Liu Y., Welch M.J. (2012). Nanoparticles Labeled with Positron Emitting Nuclides: Advantages, Methods, and Applications. Bioconjug. Chem..

[129] Ojha N., Dhamoon A.S. (2021). Myocardial Infarction. StatPearls [Internet].

[130] Huang S., Frangogiannis N.G. (2018). Anti-Inflammatory Therapies in Myocardial Infarction: Failures, Hopes and Challenges. Br. J. Pharmacol..

[131] Khan S., Hasan A., Attar F., Sharifi M., Siddique R., Mraiche F., Falahati M. (2020). Gold Nanoparticle-Based Platforms for Diagnosis and Treatment of Myocardial Infarction. ACS Biomater. Sci. Eng..

[132] Mir M., Ahmed N., ur Rehman A. (2017). Recent Applications of PLGA Based Nanostructures in Drug Delivery. Colloids Surf. B Biointerfaces.

[133] Dong F., Cui Z., Teng G., Shangguan K., Zhang Q., Zhang G. (2022). Green Synthesis of Gold Nanoparticles (AuNPs) As Potential Drug Carrier for Treatment and Care of Cardiac Hypertrophy Agents. J. Clust. Sci..

[134] Sui Y., Xie L., Meng D., Ruan Y., Zhong Z., Huang L. (2022). Cardiovascular Protective Properties of Green Synthesised Iron Nanoparticles from Calendula Officinalis Leaf Aqueous Extract on Mitoxantrone-Induced DNA Fragmentation and Apoptosis in HDMVECn, HUVEC, HAEC, HCAEC, HCASMC and HPAEC Cells. J. Exp. Nanosci..

[135] Utzinger J., Becker S.L., Knopp S., Blum J., Neumayr A.L., Keiser J., Hatz C.F. (2012). Neglected Tropical Diseases: Diagnosis, Clinical Management, Treatment and Control. Swiss Med. Wkly. Off. J. Swiss Soc. Infect. Dis. Swiss Soc. Intern. Med. Swiss Soc. Pneumol..

[136] Reuling I.J. (2021). The Use of Controlled Human Malaria Infection As Fit-For-Purpose Model In The Fight Against Malaria. https://repository.ubn.ru.nl/handle/2066/217375.

[137] Salem S.S., Hammad E.N., Mohamed A.A., El-Dougdoug W. (2022). A Comprehensive Review of Nanomaterials: Types, Synthesis, Characterization, and Applications. Biointerface Res. Appl. Chem..

[138] Obisesan O.R., Adekunle A.S., Oyekunle J.A.O., Ogunfowokan A.O., Olaniran O., Thomas S., Nkambule T.T.I., Mamba B.B. (2020). Catalytic Degradation of β-Hematin (Malaria Biomaker) Using Some Selected Metal Oxide Nanoparticles. Mater. Res. Express.

[139] Almatroudi A. (2020). Silver Nanoparticles: Synthesis, Characterisation and Biomedical Applications. Open Life Sci..

[140] Rahman K., Khan S.U., Fahad S., Chang M.X., Abbas A., Khan W.U., Rahman L., Haq Z.U., Nabi G., Khan D. (2019). Nano-Biotechnology: A New Approach to Treat and Prevent Malaria. Int. J. Nanomed..

[141] Goodman A.L., Forbes E.K., Williams A.R., Douglas A.D., De Cassan S.C., Bauza K., Biswas S., Dicks M.D.J., Llewellyn D., Moore A.C. (2013). The Utility of Plasmodium Berghei as a Rodent Model for Anti-Merozoite Malaria Vaccine Assessment. Sci. Rep..

[142] Rai M., Ingle A.P., Paralikar P., Gupta I., Medici S., Santos C.A. (2017). Recent Advances in Use of Silver Nanoparticles as Antimalarial Agents. Int. J. Pharm..

[143] Okaiyeto K., Hoppe H., Okoh A.I. (2021). Plant-Based Synthesis of Silver Nanoparticles Using Aqueous Leaf Extract of *Salvia officinalis*: Characterization and Its Antiplasmodial Activity. J. Clust. Sci..

[144] Ojemaye M.O., Okoh S.O., Okoh A.I. (2021). Silver Nanoparticles (AgNPs) Facilitated by Plant Parts of *Crataegus Ambigua* Becker AK Extracts and Their Antibacterial, Antioxidant and Antimalarial Activities. Green Chem. Lett. Rev..

[145] Krishnaraj C., Jagan E.G., Rajasekar S., Selvakumar P., Kalaichelvan P.T., Mohan N. (2010). Synthesis of Silver Nanoparticles Using *Acalypha Indica* Leaf Extracts and Its Antibacterial Activity against Water Borne Pathogens. Colloids Surf. B Biointerfaces.

[146] Krishnaraj C., Muthukumaran P., Ramachandran R., Balakumaran M.D., Kalaichelvan P.T. (2014). *Acalypha indica* Linn: Biogenic Synthesis of Silver and Gold Nanoparticles and Their Cytotoxic Effects against MDA-MB-231, Human Breast Cancer Cells. Biotechnol. Rep..

[147] Niraimathi K.L., Sudha V., Lavanya R., Brindha P. (2013). Biosynthesis of Silver Nanoparticles Using *Alternanthera sessilis* (Linn.) Extract and Their Antimicrobial, Antioxidant Activities. Colloids Surf. B Biointerfaces.

[148] Wang D., Cui L., Chang X., Guan D. (2020). Biosynthesis and Characterization of Zinc Oxide Nanoparticles from Artemisia Annua and Investigate Their Effect on Proliferation, Osteogenic Differentiation and Mineralization in Human Osteoblast-like MG-63 Cells. J. Photochem. Photobiol. B Biol..

[149] Singh S.P., Mishra A., Shyanti R.K., Singh R.P., Acharya A. (2021). Silver Nanoparticles Synthesized Using *Carica papaya* Leaf Extract (AgNPs-PLE) Causes Cell Cycle Arrest and Apoptosis in Human Prostate (DU145) Cancer Cells. Biol. Trace Elem. Res..

[150] Happy A., Soumya M., Venkat Kumar S., Rajeshkumar S., Sheba Rani N.D., Lakshmi T., Deepak Nallaswamy V. (2019). Phyto-Assisted Synthesis of Zinc Oxide Nanoparticles Using *Cassia Alata* and Its Antibacterial Activity against *Escherichia coli*. Biochem. Biophys. Rep..

[151] Al-Shmgani H.S.A., Mohammed W.H., Sulaiman G.M., Saadoon A.H. (2017). Biosynthesis of Silver Nanoparticles from Catharanthus Roseus Leaf Extract and Assessing Their Antioxidant, Antimicrobial, and Wound-Healing Activities. Artif. Cells Nanomed. Biotechnol..

[152] Velsankar K., Sudhahar S., Parvathy G., Kaliammal R. (2020). Effect of Cytotoxicity and AAntibacterial Activity of Biosynthesis of ZnO Hexagonal Shaped Nanoparticles by Echinochloa Frumentacea Grains Extract as a Reducing Agent. Mater. Chem. Phys..

[153] Ahmad W., Kalra D. (2020). Green Synthesis, Characterization and Anti Microbial Activities of ZnO Nanoparticles Using Euphorbia Hirta Leaf Extract. J. King Saud Univ.—Sci..

[154] Elumalai D., Hemavathi M., Deepaa C.V., Kaleena P.K. (2017). Evaluation of Phytosynthesised Silver Nanoparticles from Leaf Extracts of *Leucas Aspera* and *Hyptis Suaveolens* and Their Larvicidal Activity against Malaria, Dengue and Filariasis Vectors. Parasite Epidemiol. Control.

[155] Muralikrishna T., Malothu R., Pattanayak M., Nayak P.L. (2014). Green Synthesis of Gold Nanoparticles Using *Mangifera indica* (Mango Leaves) Aqueous Extract. World J. Nano Sci. Technol..

[156] Dhandapani K.V., Anbumani D., Gandhi A.D., Annamalai P., Muthuvenkatachalam B.S., Kavitha P., Ranganathan B. (2020). Green Route for the Synthesis of Zinc Oxide Nanoparticles from *Melia azedarach* Leaf Extract and Evaluation of Their Antioxidant and Antibacterial Activities. Biocatal. Agric. Biotechnol..

[157] Vankar P.S., Bajpai D. (2010). Preparation of Gold Nanoparticles from Mirabilis Jalapa Flowers. Indian J. Biochem. Biophys..

[158] Tang Q., Xia H., Liang W., Huo X., Wei X. (2020). Synthesis and Characterization of Zinc Oxide Nanoparticles from Morus Nigra and Its Anticancer Activity of AGS Gastric Cancer Cells. J. Photochem. Photobiol. B Biol..

[159] Bhau B.S., Ghosh S., Puri S., Borah B., Sarmah D.K., Khan R. (2015). Green Synthesis of Gold Nanoparticles from the Leaf Extract of Nepenthes Khasiana and Antimicrobial Assay. Adv. Mater. Lett..

[160] Velmurugan P., Lee S.-M., Iydroose M., Lee K.-J., Oh B.-T. (2013). Pine Cone-Mediated Green Synthesis of Silver Nanoparticles and Their Antibacterial Activity against Agricultural Pathogens. Appl. Microbiol. Biotechnol..

[161] Singh K., Naidoo Y., Mocktar C., Baijnath H. (2018). Biosynthesis of Silver Nanoparticles Using *Plumbago auriculata* Leaf and Calyx Extracts and Evaluation of Their Antimicrobial Activities. Adv. Nat. Sci. Nanosci. Nanotechnol..

[162] Panneerselvam C., Murugan K., Roni M., Aziz A.T., Suresh U., Rajaganesh R., Madhiyazhagan P., Subramaniam J., Dinesh D., Nicoletti M. (2016). Fern-Synthesized Nanoparticles in the Fight against Malaria: LC/MS Analysis of *Pteridium aquilinum* Leaf Extract and Biosynthesis of Silver Nanoparticles with High Mosquitocidal and Antiplasmodial Activity. Parasitol. Res..

[163] Xu Z., Wu Y., Song L., Chinnathambi A., Ali Alharbi S., Fang L. (2020). Anticarcinogenic Effect of Zinc Oxide Nanoparticles Synthesized from Rhizoma Paridis Saponins on Molt-4 Leukemia Cells. J. King Saud Univ.—Sci..

[164] Dube P., Meyer S., Madiehe A., Meyer M. (2020). Antibacterial Activity of Biogenic Silver and Gold Nanoparticles Synthesized from *Salvia Africana-Lutea* and *Sutherlandia frutescens*. Nanotechnology.

[165] Bayrami A., Alioghli S., Rahim Pouran S., Habibi-Yangjeh A., Khataee A., Ramesh S. (2019). A Facile Ultrasonic-Aided Biosynthesis of ZnO Nanoparticles Using *Vaccinium Arctostaphylos* L. Leaf Extract and Its Antidiabetic, Antibacterial, and Oxidative Activity Evaluation. Ultrason. Sonochem..

[166] Haider A., Ijaz M., Ali S., Haider J., Imran M., Majeed H., Shahzadi I., Ali M.M., Khan J.A., Ikram M. (2020). Green Synthesized Phytochemically (*Zingiber Officinale* and *Allium sativum*) Reduced Nickel Oxide Nanoparticles Confirmed Bactericidal and Catalytic Potential. Nanoscale Res. Lett..

[167] Mujeeb A.A., Khan N.A., Jamal F., Badre Alam K.F., Saeed H., Kazmi S., Alshameri A.W.F., Kashif M., Ghazi I., Owais M. (2020). Olax Scandens Mediated Biogenic Synthesis of Ag-Cu Nanocomposites: Potential Against Inhibition of Drug-Resistant Microbes. Front. Chem..

[168] Mukherjee S., Chowdhury D., Kotcherlakota R., Patra S., Vinothkumar B., Bhadra M.P., Sreedhar B., Patra C.R. (2014). Potential Theranostics Application of Bio-Synthesized Silver Nanoparticles (4-in-1 System). Theranostics.

[169] Păduraru D.N., Ion D., Niculescu A.G., Mușat F., Andronic O., Grumezescu A.M., Bolocan A. (2022). Recent Developments in Metallic Nanomaterials for Cancer Therapy, Diagnosing and Imaging Applications. Pharmaceutics.

[170] Nilghaz A., Mousavi S.M., Tian J., Cao R., Guijt R.M., Wang X. (2021). Noble-Metal Nanoparticle-Based Colorimetric Diagnostic Assays for Point-of-Need Applications. ACS Appl. Nano Mater..

[171] Sibuyi N.R.S., Moabelo K.L., Fadaka A.O., Meyer S., Onani M.O., Madiehe A.M., Meyer M. (2021). Multifunctional Gold Nanoparticles for Improved Diagnostic and Therapeutic Applications: A Review. Nanoscale Res. Lett..

[172] Bhardwaj K., Dhanjal D.S., Sharma A., Nepovimova E., Kalia A., Thakur S., Bhardwaj S., Chopra C., Singh R., Verma R. (2020). Conifer-Derived Metallic Nanoparticles: Green Synthesis and Biological Applications. Int. J. Mol. Sci..

[173] Govindaraju K., Krishnamoorthy K., Alsagaby S.A., Singaravelu G., Premanathan M. (2015). Green Synthesis of Silver Nanoparticles for Selective Toxicity towards Cancer Cells. IET Nanobiotechnol..

[174] Sabella S., Carney R.P., Brunetti V., Malvindi M.A., Al-Juffali N., Vecchio G., Janes S.M., Bakr O.M., Cingolani R., Stellacci F. (2014). A General Mechanism for Intracellular Toxicity of Metal-Containing Nanoparticles. Nanoscale.

[175] Sengul A., Asmatulu E. (2020). Toxicity of Metal and Metal Oxide Nanoparticles: A Review. Environ. Chem. Lett..

[176] Jaswal T., Gupta J. (2021). A Review on the Toxicity of Silver Nanoparticles on Human Health. Mater. Today Proc..

[177] Enrico C., Enrico C. (2021). Biophysical Interaction, Nanotoxicology Evaluation, and Biocompatibility and Biosafety of Metal Nanoparticles. arXiv.

[178] Mukherjee S., Patra C.R. (2017). Biologically Synthesized Metal Nanoparticles: Recent Advancement and Future Perspectives in Cancer Theranostics. Futur. Sci. OA.

[179] Escudero-Francos M.A., Cepas V., González-Menéndez P., Badía-Laíño R., Díaz-García M.E., Sainz R.M., Mayo J.C., Hevia D. (2017). Cellular Uptake and Tissue Biodistribution of Functionalized Gold Nanoparticles and Nanoclusters. J. Biomed. Nanotechnol..

[180] Khan S.A. (2019). Metal Nanoparticles Toxicity: Role of Physicochemical Aspects.

[181] Kumar V., Sharma N., Maitra S.S. (2017). In Vitro and in Vivo Toxicity Assessment of Nanoparticles. Int. Nano Lett..

[182] Suker D.K., Jasim F.A. (2018). Liver Histopathological Alteration after Repeated Intra-Tracheal Instillation of Titanium Dioxide in Male Rats. Gastroenterol. Hepatol. Bed Bench.

[183] Yao Y., Zang Y., Qu J., Tang M., Zhang T. (2019). The Toxicity Of Metallic Nanoparticles On Liver: The Subcellular Damages, Mechanisms, And Outcomes. Int. J. Nanomed..

[184] Medici S., Peana M., Pelucelli A., Zoroddu M.A. (2021). An Updated Overview on Metal Nanoparticles Toxicity. Semin. Cancer Biol..

[185] Rana A., Yadav K., Jagadevan S. (2020). A Comprehensive Review on Green Synthesis of Nature-Inspired Metal Nanoparticles: Mechanism, Application and Toxicity. J. Clean. Prod..

[186] Sharma A., Muresanu D.F., Patnaik R., Sharma H.S. (2013). Size- and Age-Dependent Neurotoxicity of Engineered Metal Nanoparticles in Rats. Mol. Neurobiol..

[187] Mukherjee S., Sau S., Madhuri D., Bollu V.S., Madhusudana K., Sreedhar B., Banerjee R., Patra C.R. (2016). Green Synthesis and Characterization of Monodispersed Gold Nanoparticles: Toxicity Study, Delivery of Doxorubicin and Its Bio-Distribution in Mouse Model. J. Biomed. Nanotechnol..

[188] Zhang D., Ma X.L., Gu Y., Huang H., Zhang G.W. (2020). Green Synthesis of Metallic Nanoparticles and Their Potential Applications to Treat Cancer. Front. Chem..

[189] Niska K., Pyszka K., Tukaj C., Wozniak M., Radomski M.W., Inkielewicz-Stepniak I. (2015). Titanium Dioxide Nanoparticles Enhance Production of Superoxide Anion and Alter the Antioxidant System in Human Osteoblast Cells. Int. J. Nanomed..

[190] Chibber S., Ansari S.A., Satar R. (2013). New Vision to CuO, ZnO, and TiO2 Nanoparticles: Their Outcome and Effects. J. Nanopart. Res..

[191] Song M.F., Li Y.S., Kasai H., Kawai K. (2012). Metal Nanoparticle-Induced Micronuclei and Oxidative DNA Damage in Mice. J. Clin. Biochem. Nutr..

[192] Santonastaso M., Mottola F., Colacurci N., Iovine C., Pacifico S., Cammarota M., Cesaroni F., Rocco L. (2019). In Vitro Genotoxic Effects of Titanium Dioxide Nanoparticles (n-TiO2) in Human Sperm Cells. Mol. Reprod. Dev..

[193] Ma L., Liu J., Li N., Wang J., Duan Y., Yan J., Liu H., Wang H., Hong F. (2010). Oxidative Stress in the Brain of Mice Caused by Translocated Nanoparticulate TiO2 Delivered to the Abdominal Cavity. Biomaterials.

[194] Wang Z., Wang Z. (2020). Nanoparticles Induced Embryo–Fetal Toxicity. Toxicol. Ind. Health.

[195] Campagnolo L., Massimiani M., Vecchione L., Piccirilli D., Toschi N., Magrini A., Bonanno E., Scimeca M., Castagnozzi L., Buonanno G. (2017). Silver Nanoparticles Inhaled during Pregnancy Reach and Affect the Placenta and the Foetus. Nanotoxicology.

[196] Asharani P.V., Lian Wu Y., Gong Z., Valiyaveettil S. (2008). Toxicity of Silver Nanoparticles in Zebrafish Models. Nanotechnology.

[197] Teng C., Jia J., Wang Z., Sharma V.K., Yan B. (2019). Size-Dependent Maternal-Fetal Transfer and Fetal Developmental Toxicity of ZnO Nanoparticles after Oral Exposures in Pregnant Mice. Ecotoxicol. Environ. Saf..

[198] Yang H., Du L., Tian X., Fan Z., Sun C., Liu Y., Keelan J.A., Nie G. (2014). Effects of Nanoparticle Size and Gestational Age on Maternal Biodistribution and Toxicity of Gold Nanoparticles in Pregnant Mice. Toxicol. Lett..

[199] Jing X., Park J.H., Peters T.M., Thorne P.S. (2015). Toxicity of Copper Oxide Nanoparticles in Lung Epithelial Cells Exposed at the Air-Liquid Interface Compared with in Vivo Assessment. Toxicol. Vitr..

[200] Sun X., Wang Z., Zhai S., Cheng Y., Liu J., Liu B. (2013). In Vitro Cytotoxicity of Silver Nanoparticles in Primary Rat Hepatic Stellate Cells. Mol. Med. Rep..

[201] (2012). Roshan Rezaee Ranjbar Sardari Toxicological Effects of Silver Nanoparticles in Rats. African J. Microbiol. Res..

[202] Hussain S.M., Hess K.L., Gearhart J.M., Geiss K.T., Schlager J.J. (2005). In Vitro Toxicity of Nanoparticles in BRL 3A Rat Liver Cells. Toxicol. Vitr..

[203] Mohammed Salih N.F., Al-Nakeeb G.D. (2019). Histological Comparative of Kidney of Neonatal Mice Exposed to Silver Nanoparticles during Fetal Development. Int. J. Pharm. Qual. Assur..

[204] Fatemi M., Moshtaghian J., Ghaedi K., Dinani N.J. (2017). Effects of Silver Nanoparticle on the Developing Liver of Rat Pups after Maternal Exposure. Iran. J. Pharm. Res..

[205] Recordati C., De Maglie M., Bianchessi S., Argentiere S., Cella C., Mattiello S., Cubadda F., Aureli F., D’Amato M., Raggi A. (2016). Tissue Distribution and Acute Toxicity of Silver after Single Intravenous Administration in Mice: Nano-Specific and Size-Dependent Effects. Part. Fibre Toxicol..

[206] Balansky R., Longobardi M., Ganchev G., Iltcheva M., Nedyalkov N., Atanasov P., Toshkova R., De Flora S., Izzotti A. (2013). Transplacental Clastogenic and Epigenetic Effects of Gold Nanoparticles in Mice. Mutat. Res.—Fundam. Mol. Mech. Mutagen..

[207] Zhang X.D., Wu D., Shen X., Liu P.X., Yang N., Zhao B., Zhang H., Sun Y.M., Zhang L.A., Fan F.Y. (2011). Size-Dependent in Vivo Toxicity of PEG-Coated Gold Nanoparticles. Int. J. Nanomed..

[208] Choi S.Y., Jeong S., Jang S.H., Park J., Park J.H., Ock K.S., Lee S.Y., Joo S.W. (2012). In Vitro Toxicity of Serum Protein-Adsorbed Citrate-Reduced Gold Nanoparticles in Human Lung Adenocarcinoma Cells. Toxicol. Vitr..

[209] Chen J., Wang H., Long W., Shen X., Wu D., Song S.S., Sun Y.M., Liu P.X., Fan S., Fan F. (2013). Sex Differences in the Toxicity of Polyethylene Glycol-Coated Gold Nanoparticles in Mice. Int. J. Nanomed..

[210] Fraga S., Brandão A., Soares M.E., Morais T., Duarte J.A., Pereira L., Soares L., Neves C., Pereira E., de Lourdes Bastos M. (2014). Short- and Long-Term Distribution and Toxicity of Gold Nanoparticles in the Rat after a Single-Dose Intravenous Administration. Nanomed. Nanotechnol. Biol. Med..

[211] El Ghareeb A.E.W., Hamdi H., El Bakry A., Hmela H.A. (2015). Teratogenic Effects of the Titanium Dioxide Nanoparticles on the Pregnant Female Rats And Their Off Springs. Res. J. Pharm. Biol. Chem. Sci..

[212] Ezealisiji K.M., Siwe-Noundou X., Maduelosi B., Nwachukwu N., Krause R.W.M. (2019). Green Synthesis of Zinc Oxide Nanoparticles Using *Solanum torvum* (L) Leaf Extract and Evaluation of the Toxicological Profile of the ZnO Nanoparticles–Hydrogel Composite in Wistar Albino Rats. Int. Nano Lett..

[213] Wang C., Cheng K., Zhou L., He J., Zheng X., Zhang L., Zhong X., Wang T. (2017). Evaluation of Long-Term Toxicity of Oral Zinc Oxide Nanoparticles and Zinc Sulfate in Mice. Biol. Trace Elem. Res..

